# Genetic Analysis of Human Traits In Vitro: Drug Response and Gene Expression in Lymphoblastoid Cell Lines

**DOI:** 10.1371/journal.pgen.1000287

**Published:** 2008-11-28

**Authors:** Edwin Choy, Roman Yelensky, Sasha Bonakdar, Robert M. Plenge, Richa Saxena, Philip L. De Jager, Stanley Y. Shaw, Cara S. Wolfish, Jacqueline M. Slavik, Chris Cotsapas, Manuel Rivas, Emmanouil T. Dermitzakis, Ellen Cahir-McFarland, Elliott Kieff, David Hafler, Mark J. Daly, David Altshuler

**Affiliations:** 1 Broad Institute of MIT and Harvard, Cambridge, Massachusetts, United States of America; 2 Center for Human Genetic Research, Massachusetts General Hospital, Boston, Massachusetts, United States of America; 3 Division of Hematology Oncology, Massachusetts General Hospital, Boston, Massachusetts, United States of America; 4 Department of Molecular Biology, Massachusetts General Hospital, Boston, Massachusetts, United States of America; 5 Harvard–MIT Division of Health Sciences and Technology, Cambridge, Massachusetts, United States of America; 6 Division of Rheumatology, Immunology, and Allergy, Brigham and Women's Hospital, Boston, Massachusetts, United States of America; 7 Division of Molecular Immunology, Center for Neurologic Diseases, Brigham and Women's Hospital, Boston, Massachusetts, United States of America; 8 Harvard Medical School, Boston, Massachusetts, United States of America; 9 Harvard Medical School–Partners Healthcare Center for Genetics and Genomics, Boston, Massachusetts, United States of America; 10 Center for Systems Biology and Cardiovascular Research Center, Massachusetts General Hospital, Boston, Massachusetts, United States of America; 11 Biomedical Research Institute, Brigham and Women's Hospital, Boston, Massachusetts, United States of America; 12 Department of Medicine, Harvard Medical School, Boston, Massachusetts, United States of America; 13 Department of Mathematics, Massachusetts Institute of Technology, Cambridge, Massachusetts, United Sates of America; 14 Wellcome Trust Sanger Institute, Wellcome Trust Genome Campus, Cambridge, United Kingdom; 15 Channing Laboratory and Infectious Disease Division, Department of Medicine, Brigham and Women's Hospital, Boston, Massachusetts, United States of America; 16 Department of Genetics, Harvard Medical School, Boston, Massachusetts, United States of America; 17 Diabetes Unit, Massachusetts General Hospital, Boston, Massachusetts, United States of America; Princeton University, United States of America

## Abstract

Lymphoblastoid cell lines (LCLs), originally collected as renewable sources of DNA, are now being used as a model system to study genotype–phenotype relationships in human cells, including searches for QTLs influencing levels of individual mRNAs and responses to drugs and radiation. In the course of attempting to map genes for drug response using 269 LCLs from the International HapMap Project, we evaluated the extent to which biological noise and non-genetic confounders contribute to trait variability in LCLs. While drug responses could be technically well measured on a given day, we observed significant day-to-day variability and substantial correlation to non-genetic confounders, such as baseline growth rates and metabolic state in culture. After correcting for these confounders, we were unable to detect any QTLs with genome-wide significance for drug response. A much higher proportion of variance in mRNA levels may be attributed to non-genetic factors (intra-individual variance—i.e., biological noise, levels of the EBV virus used to transform the cells, ATP levels) than to detectable eQTLs. Finally, in an attempt to improve power, we focused analysis on those genes that had both detectable eQTLs and correlation to drug response; we were unable to detect evidence that eQTL SNPs are convincingly associated with drug response in the model. While LCLs are a promising model for pharmacogenetic experiments, biological noise and in vitro artifacts may reduce power and have the potential to create spurious association due to confounding.

## Author Summary

The use of lymphoblastoid cell lines (LCLs) has evolved from a renewable source of DNA to an in vitro model system to study the genetics of gene expression, drug response, and other traits in a controlled laboratory setting. While convincing relationships between SNPs and mRNA levels (eQTLs) have been described, the degree to which non-genetic variables also influence phenotypes in LCLs is less well characterized. In the course of attempting to map genes for drug responses in vitro, we evaluated the reproducibility of in vitro traits across replicates, the impact of the EBV virus used to transform B cells into cell lines, and the effect of in vitro culture conditions. We found that responses to at least some drugs and levels of many mRNAs can be technically well measured, but vary both across experiments and with non-genetic confounders such as growth rates, EBV levels, and ATP levels. The influence of such non-genetic factors can both decrease power to detect true relationships between DNA variation and traits and create the potential for non-genetic confounding and spurious associations between DNA variants and traits.

## Introduction

Genetic mapping offers an unbiased approach to discover genes and pathways influencing disease traits and responses to drugs and environmental exposures [1]. Unlike model organisms that can be exhaustively phenotyped and readily exposed to drugs and toxins in the laboratory, there are substantial limits to the phenotypes that can be safely elicited or measured in human subjects. Thus, there would be great value in a human in vitro model that faithfully reflects both in vivo genetics and physiology while allowing for systematic perturbation and characterization in high throughput. Such a model would be particularly useful to study the function of sequence variants mapped by whole genome association studies of common human diseases that do not fall in obvious coding sequences [2]–[6], many of which are presumed to influence disease traits through subtle effects on gene regulation.

One such model system has been proposed and extensively studied: EBV-transformed lymphoblastoid cell lines (LCLs) derived from human B-lymphocytes [7]–[13]. Lymphoblastoid cell lines have long been produced as renewable sources of DNA as part of normal and diseased cohorts. Initially, LCLs derived from genotyped CEPH pedigrees [14] and HapMap participants [15] were used to identify genomic regions linked to and associated with inter-individual variation in mRNA transcript levels (these “expression” QTLs are referred in the text below as “eQTLs”) [16]–[19]. A small number of such eQTLs have been found to also be associated with human disease [20]–[22]. LCLs have also been used to search for genetic variants that predict for response to radiation and drugs in vitro [23]–[26]. Some investigators have performed joint analysis of eQTLs and drug response QTLs, seeking non-random relationships between genotypes at single nucleotide polymorphisms (SNPs), baseline mRNA levels, and response to chemotherapeutic agents [27],[28]. One recent study reported identification of eQTLs that explain up to 45% of the variation seen between individuals in cell sensitivity to chemotherapy [28].

**Figure 1:**
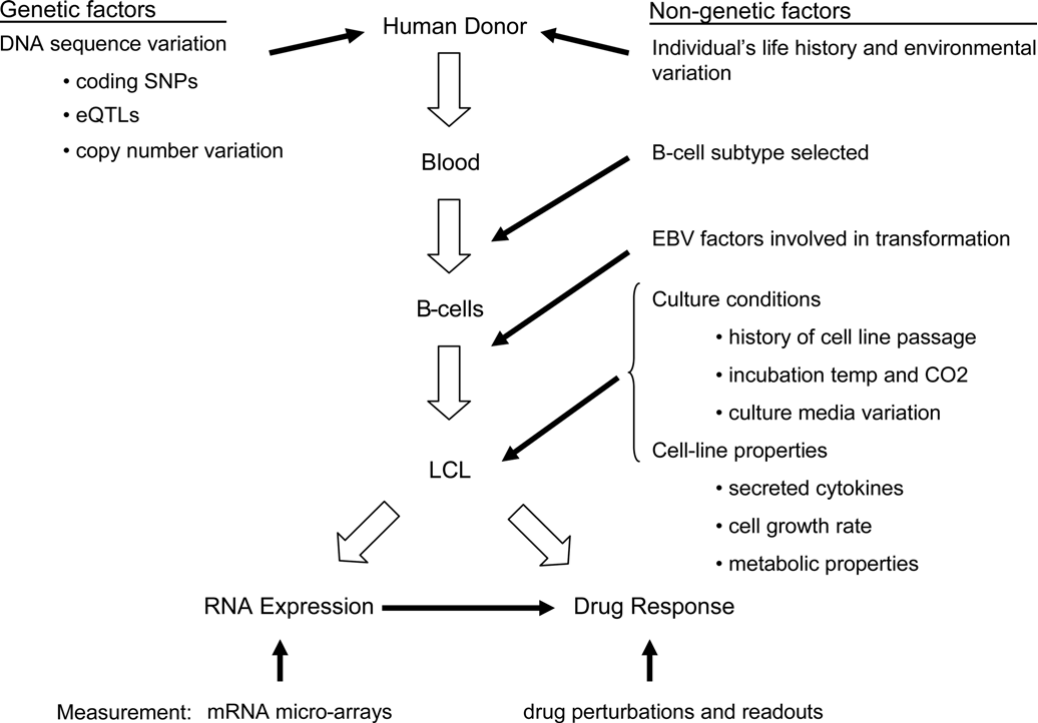
Genetic and non-genetic factors influencing lymphoblastoid cell lines as a model system to understand human physiology.

The utility of genetic mapping in LCLs is a function both of how well LCLs reflect the in vivo biology of the people from whom they were collected, and the ability to eliminate potential sources of confounding that could reduce power and cause spurious associations between cell lines (and the DNA variants they carry) and traits. While the DNA sequence of an LCL is typically a stable representation of the human donor [29], relatively less is known about the stability of cellular traits studied in vitro, and how they are influenced by non-genetic factors. Certainly, there are many opportunities for non-genetic factors to be introduced in the path from the human donor to the study of an LCL in vitro (Figure 1): the random choice of which subpopulation of B-cells are selected in the process of immortalization, the amount of and individual response to the EBV virus, the history of passage in cell culture and culture conditions, the laboratory protocols and reagents with which assays are performed, and the measurements used to assess drug response and mRNA phenotypes.

Encouraged by previous studies and the emerging HapMap resource, we set out to use LCLs to map genetic contributors to drug response in LCLs. In the course of this work we examined the relative contributions of DNA sequence variation, biological (day-to-day) variability, and confounders such as growth rate, levels of the EBV virus, ATP levels, and cell surface markers [30]. We investigated these factors in relation to two classes of phenotypes – drug response and mRNA expression levels. We find that inter-individual rank order based on both drug responses and mRNA expression levels is only modestly reproducible across independent experiments. Measurable confounders (in vitro growth rate, EBV copy number, and cellular ATP content) correlate more strongly and to a larger fraction of traits than do DNA variants. Even after correcting for confounders, and after integrating both eQTLs and mRNA correlations to drug response into a single model, we were unable to find convincing evidence for QTLs associated with drug response. Our observations suggest that, in addition to larger sample sizes, careful attention to influences of potential confounders will be valuable in the attempt to perform genetic mapping of drug responses in LCLs in vitro.

## Results

### Data Collected

We studied 269 cell lines densely genotyped by the International HapMap Project [31]. Cell lines were cultured under a structured protocol and characterized at baseline for a variety of cellular phenotypes including growth rate, ATP levels, mitochondrial DNA copy number, EBV copy number, and measures of B-cell relevant cell surface receptors and cytokine levels. Each cell line was exposed in 384-well plates to a range of doses for each of seven drugs selected based on their divergent mechanisms of action and importance in clinical use for treatment of B-cell diseases, focusing on anti-cancer agents: 5-fluorouracil (5FU), methotrexate (MTX), simvastatin, SAHA, 6-mercaptopurine (6MP), rapamycin, and bortezomib. Drug response was measured using Celltiter Glo, an ATP-activated intracellular luminescent marker that, when compared to mock-treated control wells, can represent relative levels of cellular viability and metabolic activity. Data can be downloaded from the Broad Institute web site: http://www.broad.mit.edu/mpg/pubs/hapmap_cell_lines/.

Total RNA was collected at baseline and mRNA transcript levels (hereafter referred to as “RNA”) were measured genome-wide on the Affymetrix platform. Expression data is available on GEO Accession # GSE11582. For QC and normalization details, see Materials and Methods.

Baseline characterization and plating for drug response experiments was performed in batches of 90 cell lines from each HapMap analysis panel (CEU, JPT/CHB, and YRI) on each of three experiment days. The order of cell lines within each panel was randomized to avoid inducing artificial intra-familial correlation. Each drug was tested at a range of doses around the expected IC50 as reported for the drug by the NCI DTP; each dose of drug was tested in two wells per plate and on two separate plates. These replicate measurements for each cell line allowed assessment of intra-experimental variation.

To evaluate day-to-day (i.e. inter-experimental) variation in all traits, a subset of 90 cell lines (30 from each of the three HapMap panels) was grown from freshly thawed aliquots and the entire experiment was repeated. To evaluate the effect of technical error on measured RNA levels, a set of 22 RNAs previously expression profiled (using Illumina HumanChip) at Wellcome Trust Sanger Institute (WTSI) was included in expression profiling at the Broad on Affymetrix arrays.

### Cell Line Sensitivity to Chemotherapeutic Drugs

Gene mapping of drug response (or any cellular phenotype) in LCLs requires that the phenotype be: (1) technically well measured, (2) biologically reproducible across independent experiments, and (3) remain relatively free from confounding factors. We assessed each of these characteristics in turn before performing genome-wide association scans.

To evaluate variability in drug response across replicate plates assayed on a given experiment day (technical reproducibility), we calculated the “relative” response of a cell line to each drug by measuring the (signed) distance of that cell line's dose-response curve for the drug on a given plate to the dose-response curve for the drug averaged across all cell lines assayed that day, in that replicate plate set. (The two replicate plates for each cell line performed on an experiment day were arbitrarily placed into set A or B.) This non-parametric approach allowed all drugs to be treated uniformly (see Methods) and generated two data points per cell line, per drug, per day. We ranked the cell lines based on their relative response in plate set A and separately based on values from plate set B. The rank-correlation (Spearman's rho) for relative response across sets A and B was high (rho = 0.86 to rho = 0.99, Table S1), indicating that drug response on a given day is both highly reproducible and technically well measured in this experimental design.

To evaluate variability across independent experiments on separate days (biological reproducibility), we repeated the assay on a subset of ∼90 cell lines (30 from each of the three HapMap analysis panels). (At this point, we noted that our assays for rapamycin and bortezomib suffered from weak responses and strong dependence on drug batch, respectively, and removed these drugs from future analysis; see Methods for details). For the remaining five drugs, cell lines were ranked based on relative response on day 1 and again on day 2 as above, and the rank-correlation (Spearman's rho) was calculated. In comparison to the high technical reproducibility on a given experimental day, inter-cell line variability in drug response was much less reproducible across independent experiments (rho = 0.39–0.82, Table S2).

**Figure 2:**
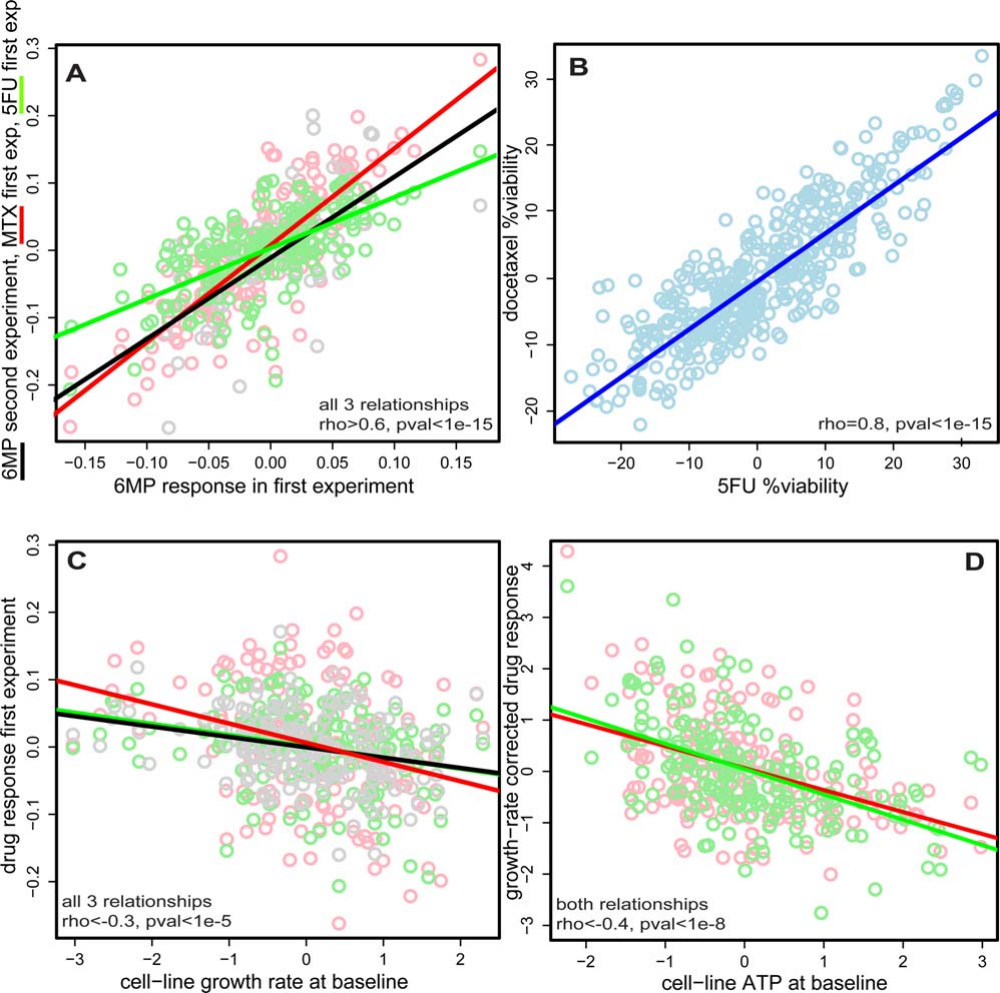
Drug response is correlated across multiple drugs, to growth rate and to baseline ATP levels of the cell line. (A) Relative drug responses were calculated for each individual as described in Methods to obtain a single number summary of the cell line response to each drug on each day. The black circles represent an individual cell line's relative response to 6MP assayed on day one plotted against 6MP relative response assayed on day two. The red circles similarly represent relative response to 6MP plotted against relative response to MTX, both assayed on day one. The green circles represent relative response to 6MP plotted against relative response to 5FU, again both assayed on day one. Lines represent regressions for each of the three comparisons and show that not only is relative drug response a reproducible trait, but also can be correlated across multiple drugs. (B) Using online data made publicly available by Watters et al. [25], relative drug response to docetaxel and 5FU was calculated using the 427 individuals with no missing data to obtain a single number for each drug, in each individual, as in (A). Response to docetaxel was plotted against 5FU for each individual. The line represents the regression for the comparison and indicates that the effect observed in (A) is neither limited to our experiments, nor to the particular drugs we attempted. (C) The baseline growth-rate of each individual's cell line was estimated as described in the Methods. This growth rate is plotted against relative response for 6MP (black), MTX (red), and 5FU (green). Lines represent regressions for the respective comparisons and all correspond to significant correlations. (D) For each individual, baseline ATP levels were measured using Celltiter glo in the mock-treated wells in drug response assays. EC50 response was calculated correcting for growth rate (see Methods). Relative ATP levels were plotted against the growth-rate corrected EC50 for MTX (red), and 5FU (green). Lines represent regression for the comparisons and indicate significant correlations.

We noted that the rank order of cell lines based on relative drug response was strikingly similar between three drugs (5FU, 6MP, and MTX). In fact, the rankings of cell lines based on these three drugs were as similar to one another as to rankings based on biological replicates of the same drug on different days (Figure 2A and Table S3). Wondering if this observation was limited to our dataset, we examined the publicly available data of Watters et al.[25] (Figure 2B). We found a very similar correlation of relative response to a distinct pair of drugs, 5FU and docetaxel, in their experiments. (This correlation likely explains why these investigators found linkage for both drugs to the same genomic locus.) Such a correlation in relative response to multiple drugs could, in theory, indicate a shared genetic mechanism common to many drugs, but it could also suggest the influence of an experimental confounder that more strongly influences drug response than does genetic variation.

We searched for and identified one such confounder: the baseline growth rate of the individual cell lines was highly correlated to the relative responses to these drugs (Figure 2C; Table S3). Growth-rate was modestly reproducible across days (rho = 0.37), with very limited evidence for heritability (h^2^ = 0.35; pval = 0.08). (We note that our study is not well-powered to detect h^2^<0.5 (Figure S1).) The dependence of drug response on growth rate in LCLs, though not previously reported, is unsurprising: all three agents depend upon cell division. Using a differential equation model of drug response accounting for the kinetics of exponential growth under exposure to drug (see Methods), we estimated a growth rate adjusted EC50 for each cell line for each of the three affected drugs. This approach removed the bulk of the correlation between drug responses and between drug response and growth rate (Table S4), though some correlation of responses persisted. Standard EC50s were fit for Simvastatin and SAHA.

Given the residual correlation across drugs, we searched for other non-genetic confounders. Baseline ATP concentrations (estimated based on the average of Celltiter glo values for all mock-treated wells, see Methods) were correlated to the growth rate adjusted EC50s for MTX and 5FU (Figure 2D). Like growth rate, ATP levels were reproducible across biological replicates (rho = 0.6) without statistically significantly evidence for heritability (h^2^ = 0.19, pval = 0.12). After further adjusting the growth rate adjusted EC50s for MTX and 5FU for ATP levels using linear regression, the correlation across drugs was nearly abrogated (Table S5).

Having adjusted for confounding due to growth rate and ATP levels, and largely eliminating correlations across drugs that were attributable to in vitro rather than inherited influences, we performed genome-wide association studies. Specifically, we examined the relationship between the EC50s for each drug and SNPs from HapMap Phase 2 with Minor Allele Frequency (MAF) >10% [32]. We did not observe any associations that surpassed genome wide significance (p-val<5e-8). The study was well powered to detect only strong QTLs, those that explain >15% of the variance in drug response (Figure S2). Nonetheless, the distributions of statistical association between SNPs and EC50s did not significantly exceed expectation under the null hypothesis. Our lack of evidence for association between SNPs and drug responses is consistent with prior publications [24]–[28], none of which identified specific SNPs that exceeded genome wide significance.

### Variability in RNA Expression

Previous studies observed baseline levels of RNA expression correlated to response to cisplatin and etoposide [24],[27],[28]. A correlation does not imply a causal contribution to drug response, as a third factor could simultaneously affect both phenotypes. Nonetheless, in the effort to identify a subset of genes whose regulation may truly influences drug response, it may be valuable to integrate information on SNP associations with RNA levels (eQTLs) and RNA correlations to drug responses. We therefore turned our attention to RNA measurements in LCLs.

As with drug response, genetic mapping of variants that influence RNA expression requires that interindividual variation in RNA levels is (a) reproducible on a given day, (b) reproducible across experiments performed on different days, and (c) influenced by genetic variation to a greater extent and independent of confounding by experimental artifacts.

**Figure 3:**
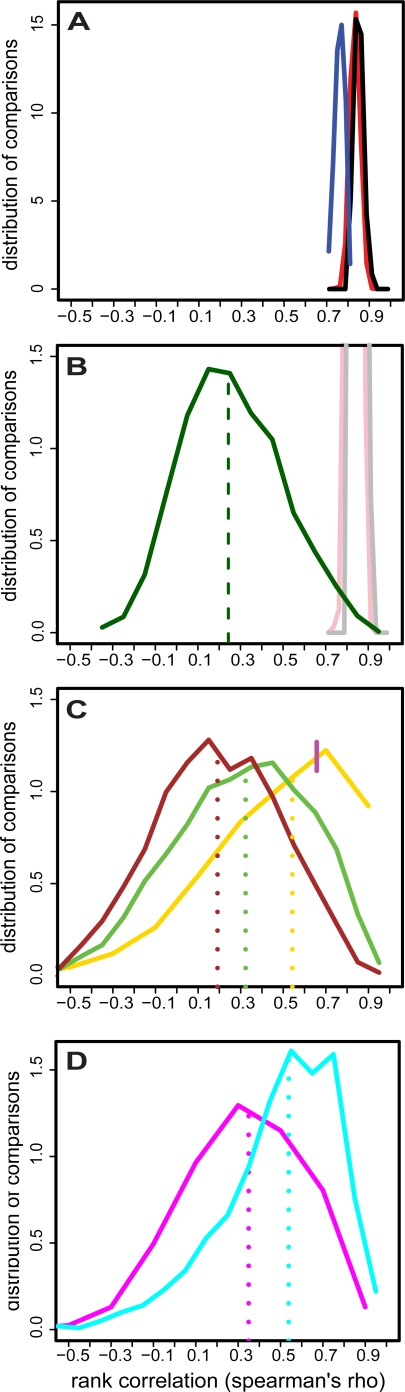
Biological variation in RNA expression. 49 unrelated individuals were whole-genome RNA profiled on the Affymetrix platform in two independent experiments at the Broad Institute. (same-platform biological replicates) A subset of 14 (of the 49) were also profiled independently at the WTSI on the Illumina platform (cross-platform biological replicates) and an aliquot of that RNA (“WTSI RNA”) was again profiled at the Broad Institute on the Affymetrix platform. (cross-platform technical replicates) (A) Expression values of all 3538 expressed genes were ranked in each of the 14 unrelated individuals in the two Broad Institute biological replicate experiments and ranks were compared between: the same individuals in two separate experiments (black); all pairs of unrelated individuals across two experiments (red); 5 chimpanzees assayed in the first experiment and all individuals assayed in the second experiment (blue). Plot shows that *overall* expression profiles in LCLs are highly similar across biological replicates, between unrelated individuals, and even across species. (B) The 49 individuals were ranked according to their relative levels of each gene in the first Broad experiment. The ranking was then independently repeated for the second Broad experiment. Ranks were compared across the two experiments for each gene and the results plotted in (green), with the median of the distribution in (dotted green). Plot shows that when any given gene is examined, there is substantial variation in the relative order of *individuals* between two independent experiments, despite the relative order of *genes* being highly stable as shown in (A). Light black and red lines are same as (A) for comparison. (C) On the set of 14 individuals, per-gene rank comparisons as in (B) are computed for: WTSI RNA assayed on the Illumina platform vs. WTSI RNA assayed on the Affymetrix platform (gold solid and dotted); WTSI RNA assayed on the Illumina platform vs. RNA extracted at the Broad Institute during the first experiment and assayed on the Affymetrix platform (brown solid and dotted); the two independent Broad experiments as in (B), (green solid and dotted). Plot shows substantial *biological* variation in the relative levels of any given gene when profiling experiments are repeated, far in excess of that might be expected from measurement error alone. Magenta dash indicates the cut-off for the 1000 “technically best-measured” genes to use in (D). (D) The analysis for the brown and green curve in (C) is repeated only for the 1000 “best-measured” genes and plotted in magenta and cyan respectively. Plot shows that even if measurement noise is limited, a substantial portion of the variance in gene expression represents biological noise.

One common metric for evaluating reproducibility in expression data is to rank the level of expression of all genes in a given sample, and to compare these ranks of genes (relative to one another) to those obtained in a separate hybridization of another aliquot of the same RNA (technical replicates) or in RNA from the same cell line on a different day (biological replicates). When we assessed the reproducibility of ranked RNA levels using this metric, we observed a high correlation across biological replicates: (Figure 3A – black curve). Moreover, we observed a similar correlation between profiles from any pair of unrelated individuals (Figure 3A – red curve), and across human cell lines in comparison to those from chimpanzee (Figure 3A – blue curve). What this reflects is the simple fact that the dynamic range in expression levels across genes is stable across primates, and much larger in magnitude than the inter-individual variation in the level of any given gene.

A more relevant metric for gene mapping is the reproducibility in rank order of different individuals based on the level of expression of a given gene. If the level of a single RNA transcript in one individual is reproducibly higher than the same RNA transcript in another individual, then it may be possible to identify genetic variants contributing to inter-individual variation of this RNA transcript (i.e. an eQTL). In contrast, if variation in the level of an RNA transcript across individuals is low relative to the technical and biological noise in a single individual, then there will be limited power to map genetic influences that alter expression of the gene.

We examined inter-individual variation in RNA levels for each of 3,538 genes measured to be expressed in the cell lines (using standard criteria for expression arrays). The analysis included LCLs from 49 unrelated individuals that were independently thawed, cultured and profiled on two different days (Figure 3B). In contrast to the results in Figure 3A, which showed excellent technical reproducibility, we see that the rank-correlation of individuals on different days (based on measured levels of individual genes) is typically modest (rho = 0.25–0.3). That is, in our experiment, only a fraction of the 3,538 RNA transcripts examined in LCLs vary reproducibly between individuals relative to technical and biological noise.

To parse the contributions of technical and biological noise, we examined the reproducibility of rank orders of cell lines when aliquots from the same RNA sample were profiled on two different array platforms. Specifically, RNAs for 14 unrelated individuals (from YRI HapMap subset) were profiled using the Illumina system at WTSI, and these same RNA samples were profiled on Affymetrix microarrays at Broad. To evaluate the contribution of technical measurement error, we calculated reproducibility in the rank order of individuals based on these technical replicates. We observed a median rank-correlation of rho = 0.55 (Figure 3C – gold curve), much higher than the biological reproducibility observed when two RNA samples for the same 14 individuals were independently prepared in a single lab and expression profiled on the same platform (rho∼0.3, Figure 3C – green curve). Thus, biological variation in RNA expression is greater than measurement error, even across different technologies.

To further minimize the impact of technical measurement error, we henceforth restricted analysis to one thousand genes that displayed the greatest technical reproducibility in rank ordering individuals (rho>∼0.7, median rho∼0.85). Genes excluded by this threshold include both those that are technically well measured but invariant across individuals, and those for which inter-individual variation is obscured by technical noise. (As the WTSI performed four technical replicates while Broad performed only a single technical replicate, WTSI data had lower overall variance.) Genes excluded by this filter typically varied less across individuals, particularly in the better-measured WTSI dataset. (median standard deviation of 1000 best-measured genes = 0.27 vs 0.17 for the other ∼2500 expressed genes; p-val<1e-15).

When analysis was limited to these one thousand genes, the correlation across biological replicates improved but was still modest (rho = 0.55, Figure 3D – cyan). That is, despite excellent technical reproducibility overall (Figure 3A) even relative to inter-individual variation (Figure 3C), the rank order of individuals based on most genes was only partially reproducible.

**Figure 4 pgen-1000287-g004:**
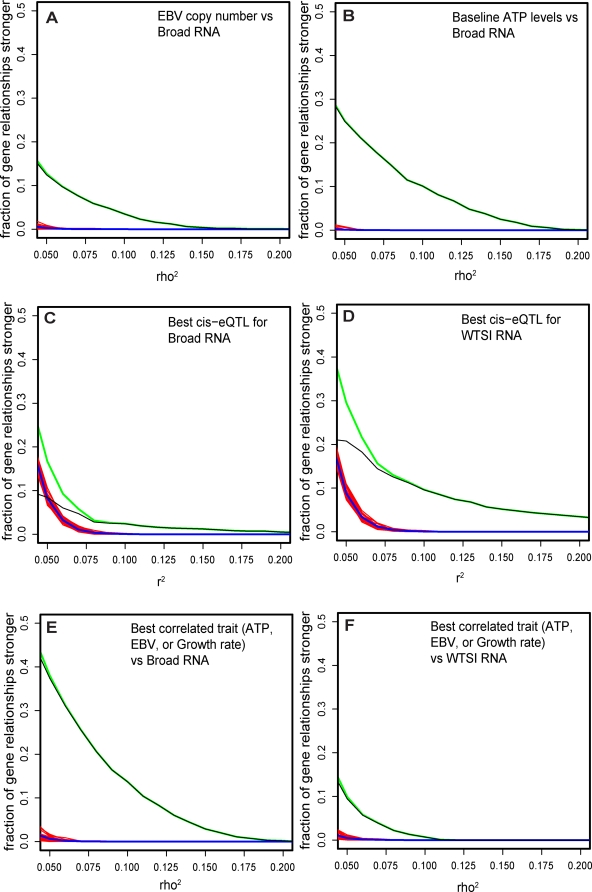
RNA expression is correlated to SNPs and cellular traits. 198 unrelated individuals were whole-genome RNA profiled on the Affymetrix platform at the Broad Institute (“Broad RNA”) and independently on the Illumina platform at WTSI (“WTSI RNA”). The 1000 “best-measured” genes identified in Figure 3 were tested for correlation to SNPs and cellular traits. (A) For each tested gene, Broad RNA expression levels were rank-correlated to copy numbers of EBV, as determined by quantitative PCR. The correlation was expressed as rho^2^ and curves representing distributions of the rho^2^values are plotted. The green curve is the observed distribution of EBV-RNA correlations. The red curves represent 20 permuted distributions. The blue curve is the average of permuted distributions. The black curve is the difference between observed and permuted values and thus a lower bound (see Methods) of the fraction of genes correlated to EBV at a given rho^2^. Plot shows that ∼15% of expressed genes have >5% of their (rank) variance in expression explained by EBV levels. (B) For each tested gene, Broad RNA expression levels were correlated to baseline ATP levels determined by measuring Celltiter glo in mock-treated wells in the drug response assays. Curves representing the distribution of rho^2^ values were plotted for the tested genes as in (A). Plot shows that >25% of expressed genes have >5% of their variance in expression explained by ATP levels. (C) For each tested gene, Broad RNA expression levels were correlated to all SNPs with MAF>10% within a 0.15 Mb window around the gene, using the HapMap phase II data. Curves representing the distribution of the largest r^2^ value was plotted for each tested genes as in (A). Plot shows that >9% of genes have >5% of their variance in expression explained by SNPs in the Broad RNA dataset. (D) For each tested gene, Sanger RNA expression levels were correlated to all SNPs with MAF>10% within a 0.15 Mb window around the gene, using the HapMap phase II data. Curves representing the distribution of the strongest r^2^ value was plotted for each tested genes as in (C). Plot shows that >20% of genes have >5% of their variance in expression explained by SNPs in the WTSI RNA dataset. (E) For each tested gene, Broad RNA expression levels were correlated to EBV, growth rate, and relative ATP, and the strongest observed correlation among the 3 phenotypes was plotted. Strikingly, plot shows that >40% of genes have >5% of their variance in expression explained by one of these covariates. (F) For each tested gene, WTSI RNA expression levels were correlated to EBV, growth rate, and relative ATP, and the strongest observed correlation among the 3 phenotypes was plotted. Strikingly, plot shows that the effect of covariates in (E) is observable even when looking at a completely separate expression experiment, performed independently of covariate collection.

We reasoned that some of the biological noise might be due to other measured factors, as had been the case for drug response. Using a threshold of 5% variance explained, growth rate was correlated to levels of expression of only relatively few genes (<5%). In contrast, ∼15% of genes showed correlation to EBV copy number (Figure 4A), some of which encode genes known to participate in transduction pathways downstream of EBV signaling [33],[34],[35]. Moreover, the level of expression of >25% of genes was correlated to ATP levels (Figure 4B). In total, over 40% of genes have at least 5% of their variation in RNA levels correlated to one of three confounders above (Figure 4E).

The correlation of RNA levels to such factors could, in principle, represent intrinsic characteristics of each LCL (which could potentially be due to inherited DNA sequence variation, acting indirectly through susceptibility to EBV infection or inducing a metabolic state). Alternatively, growth rate, EBV infection, and metabolic state could represent experimental artifacts that obscures genetic contributions to gene expression variation. Interestingly, measurements of EBV copy number, ATP level, and growth rate at Broad correlate to levels of RNA expression generated independently at WTSI [18],[19] (Figure 4F), albeit more weakly than for the expression profiles generated on the same samples at the Broad. Thus, these confounders display a component intrinsic to each cell line, as well as a substantial component that is not a reproducible attribute of the cell line.

To examine how much of the variability in gene expression might be demonstrably attributed to inherited DNA variation, we searched for cis-eQTLs associated with RNA expression levels in our experiment. Using HapMap Phase 2 SNPs with MAF>10% that lie within a 0.15 Mb window around each gene, we performed standard linear regression between expression values of that gene and SNP genotypes coded 0,1,2 (representing the number of minor alleles carried by the individual). In our dataset, ∼9% of genes harbored a cis-eQTL that explained 5% or more of the gene's variance in expression levels (Figure 4C, reporting the excess of genes compared to permuted datasets). Even more eQTLs were evident in the WTSI expression data (which, due to the use of four technical replicates, has lower technical noise): >20% of genes were associated with a SNP that explains 5% or more of the variance (Figure 4D).

Consistent with previous analyses [16],[17],[18], in both data sets only a small fraction of genes displayed a cis eQTL that explained a large proportion of variance in RNA levels. Moreover, the fraction of genes that showed correlation to growth rate, EBV, and ATP substantially exceeded the fraction associated with a cis-eQTL of the same strength (compare figure 4E to 4C).

### Inter- and Intra-Individual Variance Component Analysis

**Figure 5 pgen-1000287-g005:**
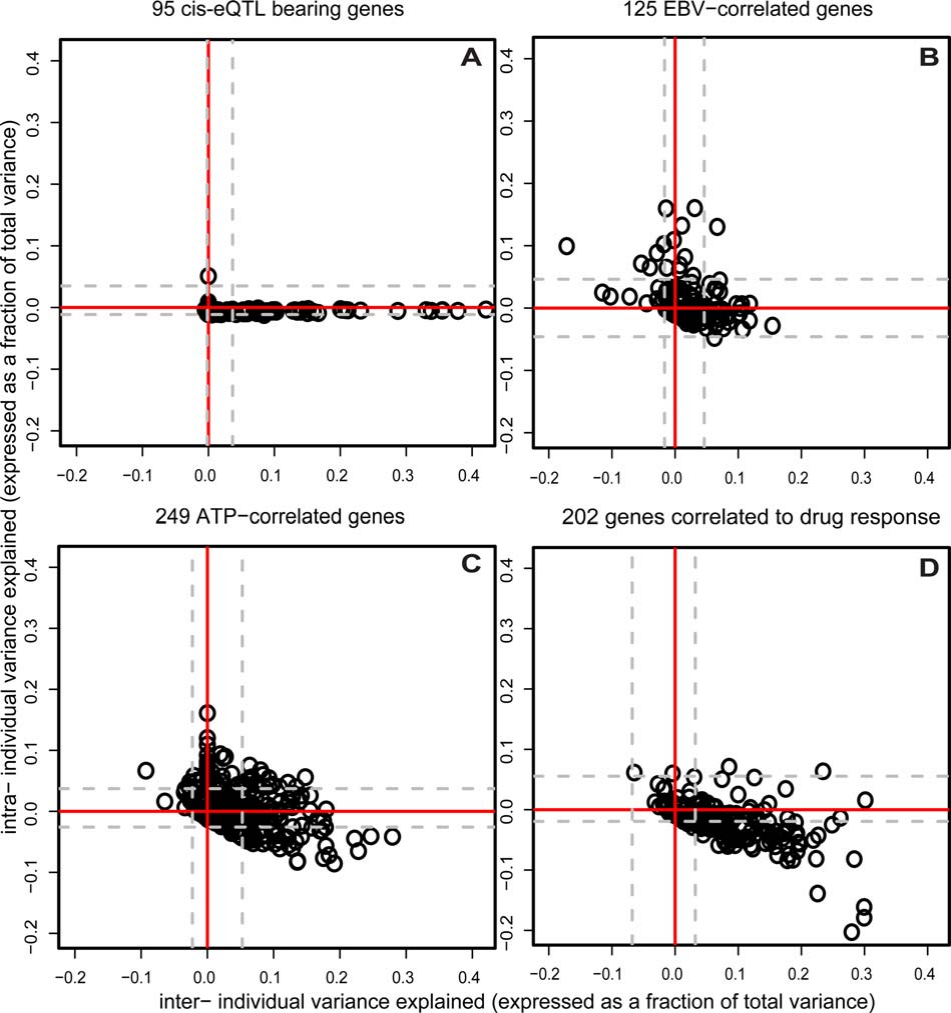
Correlation of eQTLs, EBV, and ATP to inter- and intra-individual variation in RNA expression levels, and correlation of RNA expression levels to inter- and intra-individual variation in drug response. Total variance for each of the 1000 “best-measured” genes was separated into inter- and intra- individual variance components (see Methods) using expression data from the 49 unrelated individuals measured twice at the Broad Institute on the Affymetrix platform. (A) 95 genes with eQTLs that explained >10% of expression variance (FDR<10%) in the WTSI dataset were selected (to maximize eQTL detection power) and the SNP genotype was included in the variance components model of the gene to “account” for its effect. −1 times the change in each variance component is plotted for each gene. As expected, the plot shows that that SNPs (which remain fixed across experiments) only explain inter-individual variation in expression. Grey dashed lines indicate the inter- and intra- 2.5% and 97.5%-tiles of the distribution of variance component change estimates when the entire analysis is repeated on a permuted dataset. (B) 125 genes correlated to EBV at rho^2^>.05 (FDR<10%) were selected and the EBV measurement was included in the variance components model of the gene to “account” for its effect. −1 times the change in each variance component is plotted for each gene. The plot shows that EBV is correlated to inter-individual differences in gene expression that persist across experiments, intra-individual fluctuation in gene expression between experiments, or both, depending on the gene in question. Grey dashed lines are as in (A). (C) 249 genes correlated to ATP at rho^2^>.05 (FDR<10%) were selected and the ATP measurement was included in the variance components model of the gene to “account” for its effect. −1 times the change in each variance component is plotted for each gene. The plot shows that ATP is correlated to inter-individual differences in gene expression that persist across experiments, intra-individual fluctuation in gene expression between experiments, or both, depending on the gene in question. Grey dashed lines are as in (A). (D) 202 “drug-response correlated” genes were defined as in Figure 6. The expression of each gene was incorporated in a variance components model of the assigned drug response EC50 to examine the correlation of the gene to its strongest correlated drug. −1 times the change in the variance components of drug response is plotted for each gene, showing that it is mostly the *inter*- individual differences in gene expression that are correlated to cell line drug response. Grey dashed lines are as in (A).

To parse the association of SNPs and other measures with variation in gene expression, we decomposed the total variance in expression of each gene into inter-individual and intra-individual (experimental) variation. As expected, eQTLs contribute only to inter-individual variation (Figure 5A), while EBV and ATP are correlated to either inter-individual or intra-individual variation, depending on the gene (Figure 5B and 5C).

Taken together, these observations have a number of implications: First, RNA levels for more genes are correlated to the measured non-genetic cellular factors than are associated with individual cis-eQTLs. Second, these non-genetic factors may influence gene expression not only by varying across cell lines in a reproducible manner (like SNPs), but also by varying across experiments for the same cell line. Third, for some genes, a given non-genetic factor is correlated to inter-individual variation (genes arrayed along the x-axis in Figure 5), and yet for other genes that same factor is correlated only to intra-individual variation (genes arrayed along the y-axis). Factors correlated to inter-individual variation could, in principle, represent processes related to the action of a genetic variant, whereas those that only vary across experiments represent noise with respect to genotype-phenotype association.

### Correlation of RNA Levels to Drug Response

We observed a large number of genes whose level of RNA expression at baseline was correlated to drug response. Levels of RNA transcripts for 20% of genes in the Broad Institute dataset and 18% in the WTSI dataset were correlated (at a rho^2^>0.05) to EC50 for at least one of the drugs assayed (after growth-rate and ATP adjustment). EC50s for SAHA and 5FU appeared to have the strongest relationship to RNA levels, correlating to 8.7% and to 7.7% of genes measured at the Broad and WTSI, respectively.

Applying the variance components analysis to see how inter- and intra- individual variation in growth-rate and ATP adjusted EC50s are potentially influenced by RNA levels (and “assigning” to a given gene its strongest correlated drug), we observed that RNA levels are predominantly correlated to inter-individual differences in EC50s (Figure 5D). Much less of the correlation between RNA expression and EC50s reflects intra-individual variation. This observation supports the hypothesis that interindividual variation in RNA levels due to eQTLs may contribute to variation in drug response.

### Integrating Data from eQTLs and Drug Response in LCLs

Having evaluated SNP associations with RNA levels (eQTLs), and the correlation of RNA levels to drug response, we asked whether the two relationships might point to eQTL SNPs associated with drug response. First, we asked whether there was an enrichment of genes both correlated to drug response and associated with an eQTL. Second, for the subset of genes with both an eQTL and correlation of RNA levels to drug response, we asked whether the eQTL SNPs were associated with drug response. Finally, we evaluated whether the strength of SNP association with RNA levels (eQTL) is correlated to the strength of SNP association with drug response. None of these analyses strongly supported an influence of eQTL SNPs on drug response.

We first examined the fraction of genes whose expression is associated with an eQTL and correlated to drug response. As seen in Figure 4, ∼14% and 4.5% of genes have cis-eQTLs (r^2^>0.08, FDR<10%) in the WTSI and Broad Institute datasets respectively. In the same data, levels of RNA of 18% (WTSI) and 20% (Broad) of genes are correlated to drug response (rho^2^>0.05, FDR<10%). When we consider the intersection of eQTL-bearing genes and drug-response correlated genes in each dataset independently, however, we see that only 1.4% (WTSI) and 0.9% (Broad) of genes are both correlated to drug response and bear a cis-eQTL. Neither intersection contains more genes than would be expected by chance alone and, at most, only a small fraction of genes are involved.

**Figure 6:**
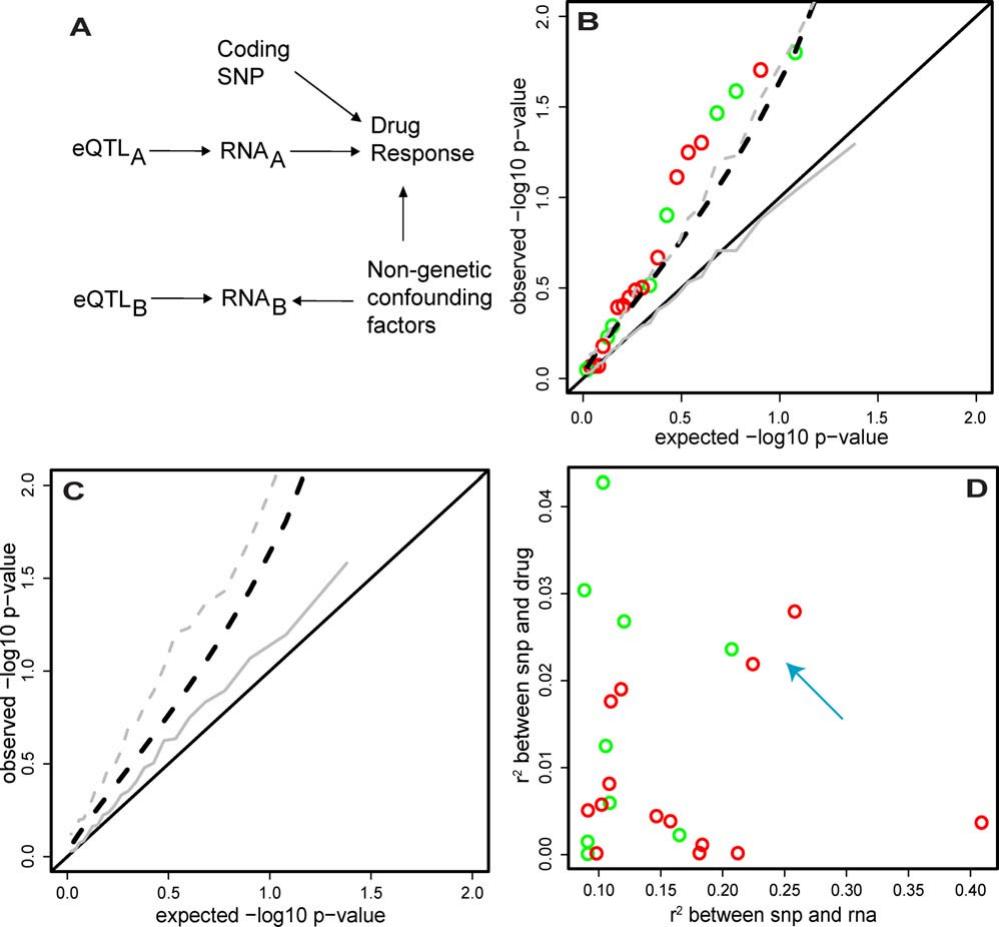
Effect of cis-eQTLs in drug-response correlated genes on drug-response. The 198 unrelated individuals were ranked by RNA expression value for each of the 1000 “best-measured” genes. These individuals were then ranked by response (growth/ATP- corrected EC50) to each of the 5 assayed drugs. Rank-correlations (spearman's rho) were computed for each gene-X-drug pair (1000×5) and the drug with the strongest correlation to a given gene was “assigned” to that gene. The 202 genes whose strongest drug correlations exceeded rho^2^ = .05 (FDR<10%) were taken as “drug-response correlated” genes. If such a gene also had a cis-eQTL that explained at least 8% (FDR<10%) of its variance, the SNP-RNA-Drug relationship was considered in the foregoing panels. We considered 23 SNP-RNA-Drug response relationships (14 derived using WTSI RNA dataset+9 derived using the Broad Institute RNA dataset). (A) Diagram of different relationships between SNPs, RNA levels, and drug response. Coding SNPs have direct (non-RNA mediated) effects on drug response by altering protein function. No SNPs of this class were found at genome-wide significance in our GWAS scan. Changes in RNA_A_ influences drug response. An eQTL for one of these RNAs (i.e. eQTL_A_) is thereby associated with drug response.Non-genetic confounding factors simultaneously influence RNA_B_ levels and drug response; changes in RNA_B_ do not influence drug response (this is the expected scenario for most RNAs). Even if levels of these RNAs are associated with eQTLs, these eQTLs are not associated with drug response. (B) For each SNP-RNA-Drug response relationship (WTSI – red, Broad – green) the drug response was regressed against the eQTL SNP genotype. P-values are plotted as open circles against their expectation under the null distribution. Black solid line indicates the theoretical flat uniform distribution expected under the null and black dashed line is the p = .05 one-sided significance threshold for deviation from the null. Grey lines show equivalent null parameters, but derived from a simulated dataset with the same SNP/RNA/Drug variances and independent SNP-RNA/RNA-Drug pairwise covariances as the real 23 SNP-RNA-Drug response relationships. Plot shows that the observed p-value distribution for drug-response regressed against RNA eQTL SNPs exceeds that expected by chance. (C) For each SNP-RNA-Drug response relationship, simulated datasets were created with the same SNP/RNA/Drug variances and RNA-Drug pairwise covariance as the real 23 SNP-RNA-Drug response relationships, but with the real SNP-RNA covariances replaced by r^2^ = 0.05. Then, only those simulations where the observed SNP-RNA association exceeded r^2^ = 0.08 were used to plot the median and p = .05 SNP-Drug p-value distributions as in (B) (again, grey solid and grey dashed lines, respectively). Black lines also as in (B). Plot shows that “winner's curse” in eQTL discovery leads to an inflation of SNP-Drug associations, in the absence of any RNA influence on Drug response. (D) For each SNP-RNA-Drug response relationship (WTSI – red, Broad – green), the correlation between SNP and RNA is plotted against the correlation between SNP and Drug. Most increased association between SNP and Drug response comes from the weaker eQTLs, while most of the stronger eQTLs have no association with drug response, consistent with the winner's curse phenomenon displayed in (C). Additionally, 3 SNP-RNA-Drug response relationships emerge that are both relatively strong SNP-RNA and SNP-Drug response associations, indicated by the light blue arrow.

Among the 1000 “best-measured” genes in each RNA dataset, we identified a total of 23 genes that happened to contain both an eQTL and showed correlation of RNA levels to drug response. We asked whether these 23 eQTL SNPs showed a non-random distribution of association with drug response. When we first regressed the drug EC50 against genotype for each of the 23 SNPs above, we saw an excess of association over that expected under the null distribution (Figure 6). Moreover, the associations of SNPs with drug response appear to be in the direction predicted by the pair-wise SNP-RNA and RNA-Drug response relationships (Figure S3). A simulated dataset with the same SNP/RNA/Drug variances and independent SNP-RNA/RNA-Drug pairwise covariances (i.e. the eQTL_B_/RNA_B_ scenario in Figure 6A) as those observed fails to demonstrate the excess association between SNPs and drug-response (Figure 6B – gray lines). Though no highly significant examples were documented, these observations are consistent with the existence of eQTLs associated with drug response (i.e. the eQTL_A_/RNA_A_ scenario in Figure 6A).

While Figure 6B might suggest that many eQTLs are associated with drug response, we recognized a potential bias that might inflate this association in absence of (or in addition to) real signal: the “winner's curse” [36] overestimate of effect size incurred during discovery of eQTLs (Figure S4). To examine this possible source of spurious association, we replaced all simulated eQTL effects in (Figure 6B) with an eQTL whose true effect is r^2^ = 0.05, but whose observed effect in simulated datasets is r^2^>0.08. In this (more realistic) simulation, we recreate an inflation of p-values similar to that observed. (Figure 6C). This analysis suggests that winner's curse may contribute to the apparent excess of association in Figure 6B.

Finally, if these eQTLs were truly influencing drug response, one might expect that stronger eQTLs would have stronger associations with drug response. We plotted the strength of each eQTL against the strength of association between the eQTL SNP and drug response. Counter to expectation, the strongest associations between SNPs and drug response are observed for SNPs that are weak eQTLs, while most of the stronger eQTLs have no association with drug response (Figure 6D). We do observe three SNPs with relatively strong drug response *and* RNA levels association (Figure 6D blue arrow): rs1384804-C8orf70 (Ensembl:ENSG00000104427)-MTX, rs3733041-GLT8D1 (Ensembl:ENSG00000016864)-5FU, and rs2279195-SH3TC1 (Ensembl:ENSG00000125089)-Simvastatin with SNP-Drug p-values of 0.03, 0.05, and 0.02 respectively. While these may be interesting candidates for follow-up and replication, statistical significance is extremely weak, and thus much larger sample sizes are required to achieve genome-wide significance.

## Discussion

Recent studies have shown that a subset of genes contain cis-eQTLs that explain a modest fraction of inter-individual variation in RNA levels. Other studies used LCLs to perform linkage and association scans for drug response [26],[27],[28]. However, few reports characterize the biological reproducibility of these phenotypes, and none to our knowledge have characterized their correlation to in vitro measures such as growth rates, EBV copy number, and metabolic activity. We document that most traits we studied, whether drug responses or RNA transcript levels, are only partially reproducible across experiments, and that more genes are correlated to cellular growth rate, ATP levels, and EBV copy numbers than to genetic variants (at comparable fractions of variance explained). Thus, in addition to issues of statistical power relative to genetic size of effect (Figure S2), day to day variability in a trait and confounding factors are major influences on gene mapping experiments in LCLs.

Consistent with prior reports, our genome-wide association studies of drug response did not reveal any SNPs associated with drug response with genome-wide significance. The inability to detect such SNPs is likely due to lack of power to detect weak effects with limited sample size (Figure S2) and in the presence of significant confounding and noise.

Several studies attempted to improve power to discover SNPs associated with drug response [15],[16] by integrating eQTLs and RNA correlations to drug response [18],[19]. Whether these eQTLs are incidental or actually contributing to drug response depends on whether the cognate RNAs influence drug response or are merely correlated to drug response by a non-genetic factor that simultaneously affects both phenotypes. Our results fail to show convincing association of eQTL SNPs with drug response (EC50s adjusted for growth rate and ATP levels). Moreover, some apparent association can be attributed to “winner's curse” (a bias possibly avoidable in the future with the creation of large cohorts for eQTL discovery). We do observe three potential associations that may merit future study: rs1384804 near C8orf70 to MTX, rs3733041 near GLT8D1 to 5FU, and rs2279195 near SH3TC1 to Simvastatin.

The hallmark of genetic mapping is causal inference: the interpretation that genetic variants at a particular genomic locus are influencing a trait of interest. This interpretation requires confidence that the association between genetic variation and phenotype is not due to confounding, but rather represents a causal relationship. In an experimental cross, causal inference is supported by meiotic randomization and the shared parents of all offspring. In a genome-wide association study, causal inference can be supported if the genomic background of study participants is observed to be null distributed and potential confounders are eliminated. Our data suggest that GWAS of LCLs need to carefully consider the major impact of non-genetic confounding in relation to the documented effects of eQTLs. In addition to reducing power, confounding by non-genetic factors can cause spurious associations between cell lines and phenotypes, violating the conditions under which causal inferences can be made.

A major limitation of our study is the relatively small sample size of the HapMap samples for performing genome-wide association studies. As much larger collections of LCLs (such as those proposed to study cell lines from eight thousand and one-hundred thousand individuals by the Framingham Heart Study [37] and the National Children's Study [38], respectively) are currently being collected, we are optimistic that larger studies have potential to map pharmacogenetic loci in LCLs. By highlighting these aspects of the LCL model, as well as pointing to how some of them may be addressed, we hope to build a stronger foundation on which these important experiments can be planned and carried out.

## Materials and Methods

### Cell Culture

EBV-transformed lymphoblastoid cell lines were acquired from the NHGRI Sample Repository for Human Genetic Research in frozen aliquots. Cells were thawed in 5 mL culture medium (RPMI medium 1640 (Invitrogen) supplemented with 10% FetalPlex (Gemini), 2 mM L-Glutamine (Invitrogen), and 1× penicillin/streptomycin (Invitrogen)). Cell lines were counted daily using Z2 Coulter Counter (Beckman Coulter) and passaged as needed to maintain a concentration of 2–5×1e5 cells/ml at 37 C in a 95% humidified 5% CO2 atmosphere.

Initially, cells were grown until 5×1e5 cells/ml were reached in 50 mL total volume. Then, ten identical aliquots were frozen in 1 mL freezing media containing 50% FetalPlex, 40% RPMI 1640 medium, and 10% DMSO (Sigma) at −80 C for 24 hrs and transferred to liquid nitrogen. These aliquots were used to provide biologic replicates for the experiments described below.

Aliquots were thawed on experiment day #1 as described above. Cell lines were counted daily and passaged as need to maintain a concentration of 4–8×1e5 cells/ml in 10 mL culture medium. On experiment day #7, cells were counted and distributed for use in the various experiments described below. One cc of culture was used for immediate immunophenotyping via FACS and Luminex beads. One cc of culture was used for RNA and DNA extraction using Trizol (Invitrogen) following the manufacturer's protocol. The remaining eight cc of culture were used for drug response assays described below.

### Drug Response Assay

The drugs that we studied are bortezomib (courtesy of T. Hideshima), rapamycin (Biomol), 5-fluorouracil (Sigma), methotrexate (Sigma), 6-mercaptopurine (MP Biomedicals), SAHA (Biovision), and simvastatin (Calbiochem). These drugs were arrayed in a source plate in the concentrations according to supplemental figure. The source plate was pinned into each cell line in duplicate, resulting in each drug concentration being assayed in each cell lines 4 times.

For drug response assays, LCLs for each cell line were diluted to 1×1e5 cells/ml, and 25 uL of cell culture were plated into each well of two white solid flat bottom 384 well plates (Corning cat# 3704) using a microplate dispenser (Multidrop Combi, Thermo Scientific). Next, 100 nL was pin-transferred from the source plates into the plates containing cells using an automated 384 channel simultaneous pippettor (CyBi-Well, CyBio). Plates were incubated at 37 C in a 95% humidified 5% CO2 incubator.

After 48 hrs, plates were removed from the incubator to room temperature for 10 minutes prior to being vortexed for 30 seconds. 25 uL of Celltiter Glo (Promega Cat No. G7573) diluted 1∶3 in PBS was added to each well with the Multidrop microplate dispenser and shaken for two minutes. Luciferase luminescence was then immediately measured for each well using a multiplate illuminometer (Envision, Perkin Elmer). Raw luminescence data is available online: http://chembank.broad.harvard.edu/assays/view-project.htm?id=1000477.

The experiment was monitored for cell-culture handling, plating, pinning, and assay errors and failed cell lines/plates/drug-rows were excluded from down-stream analysis. (Most cell lines were successfully assayed on two plates for all drugs, however; specific counts are below.) Luminescence values in drug-exposed wells were divided by the median control-well luminescence in the same plate row (after excluding plate edge wells) to obtain 4 viability fractions per cell line, per drug, per dose, in each experiment. For evaluation of technical reproducibility, the median of the 2 fractions on each plate was taken as the cell line's response to that dose on that plate. For evaluation of biological reproducibility and all other analyses, the median of the 4 fractions was taken as that cell line's response to that dose in the experiment. Drug responses were examined, and it was noted that the experiment failed to achieve meaningful cytotoxic response to rapamycin, with most cell lines reaching a maximum fractional viability of only ∼0.6–0.7, even at highest concentration of drug assayed. It was concluded that the viability assay was not a relevant read-out for rapamycin response, and the drug was not considered in further analyses.

Overall cell line response to a given drug was then calculated by taking the average response to a dose across all cell lines in the experimental batch (cell lines were assayed in batches of ∼90), subtracting the average from the value for each cell line, and then averaging the result for each cell line across all doses. (The 4–5 low-concentration doses where all cell lines had a fractional viability of ∼1 were excluded from the calculation.) In this way, the (single value) relative response of a given cell line to a drug was calculated, representing the non-parametric distance of that cell line's dose-response curve to the average dose-response curve for that drug in the experiment. (For the analysis of technical reproducibility, the calculation was done using only replicate plate A for all cell lines, and then using only replicate plate B, and the two values were compared). Quality control then proceeded by examining the dependence of response on the compound stock plate from which the drugs were pinned. (Compound stock plates were prepared with enough drug to run ∼20 cell lines and drug response should be independent of the drug stock.) Indeed, it was noted that for 5FU, 6MP, Simvastatin, SAHA, and MTX, dependence on drug stock was weak, while for bortezomib, the dependence was profound, with large differences in response between different plates, significantly in excess of the differences between cell lines on a given plate. Thus, bortezomib was excluded from further analysis. Though dependence on compound plate for the other 5 drugs was weak, average response for each compound stock plate was subtracted from each cell line using that plate (for each drug independently) and this normalized response was carried forward.

In summary, after the processing steps above in the main batch of experiments, 254 cell lines were successfully assayed for response to 6MP, 256 for MTX, 260 for Saha, 262 for Simva, and 259 for 5FU. 84 cell lines were then again successfully measured for all 5 drugs as biological replicates. (For ease of comparison, technical reproducibility is also reported using only the two plates from these biological replicate samples.) These values are available as “relative responses” in the online supplement. Analyses in Figure 2 use this data for the ∼200 successfully measured unrelated individuals, after again centering within each HapMap panel. Also, the median (non-boundary) control well luminescence over the two plates for each cell line was taken as the “ATP content” of the cell line. The value was divided by 100,000 and centered within each HapMap panel.

### Modeling Drug Response

To account for the effect of growth-rate on response to MTX, 5FU, and 6MP, we reasoned as follows: Assume a simple ODE model of cell line population growth: 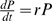, where P(t) is the # of cells in the population at a given time, and r is the (unobserved in the specific drug-exposure experiment) growth rate parameter. This ODE has the solution: *P*(*t*) = *P*_0_ *e^rt^*. When the cell line is exposed to drug, its growth-rate is impaired in a concentration-dependent manner. Taking inspiration from first-order Michaelis-Menten kinetics, we can model this as: 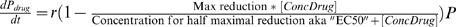, which is solved by 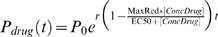. As our observed luminescences are ratios between drug wells and control wells at given concentrations, we can write 
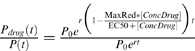
, which can simplified as 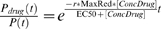.

There are two identifiable parameters in this model: the concentration necessary for half-maximal reduction in growth-rate (EC50) which is independent of growth rate r itself, and r * maximal reduction of r, a product term dependent on growth rate whose components cannot be independently estimated. The model was fit for each cell line, for each drug independently, using median measurements at all doses. QC was performed by excluding all models with RSS>0.08. The –r*MaxRed term was discarded, and the EC50 was carried into further analysis after centering the values within each HapMap panel. (257 cell lines were successfully fit for 5FU, 251 for 6MP, and 255 for MTX.) Models were also successfully fit to all 84 biological replicates of 6MP and 5FU, and 82 replicates of MTX. ATP correction for 5FU and MTX was then carried out by taking the residuals of the linear regression DRUG∼ATP.

SAHA and Simvastatin were modeled by a standard sigmoid[39], with response (fractional viability) at a given 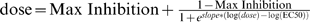. Notably, max inhibition and EC50 are *not* the same as above, here representing a minimal viability and the concentration at which that minimal viability is achieved, respectively. Maximum inhibition (aka minimum viability) were <0.05 for most cell lines for simvastatin and varied between ∼0.1–0.3 for SAHA. The EC50 was carried into further analysis after centering the values within each HapMap panel. Again, QC was performed by excluding all models with RSS>0.08. (257 cell lines were thus successfully fit for Saha and 261 for Simvastatin.) Models were also successfully fit to all 84 biological replicates of Saha and 5FU, and 83 replicates of Simvastatin. The GWAS for drug response was performed with all successfully measured individuals, while analyses presented in Figures 2,5,6 were performed with unrelated individuals only.

### Growth Rate Measurements

Each cell line was seeded at a concentration of 2×1e5 cell/mL in 2 mL. LCLs were counted daily for five consecutive days with an automated particle counter (Z2 Coulter Counter, Beckman Coulter). A regression of the form log(conc day i) = r*i+log(conc day 0) was fit for each cell line to obtain the estimate of growth rate *r*. QC was performed by evaluating the 95% confidence interval of the *r* estimate and rejecting estimates whose interval width exceeded 1.1. Thus, estimates of growth-rate for 237 cell lines were obtained. These values were normalized within each population for all analyses. An abbreviated second replicate of the experiment was repeated on a subset (155) of the cell lines with only the 3^rd^ day counts collected to evaluate growth rate reproducibility.

### FACS Analysis

From each LCL, ∼25,000 cells were incubated with R-Phycoerythrin–conjugated mouse anti-human antibody to cell surface markers (CD19, CD20, CD21, CD40, CD58, CD80, CD86, CD95, CD227, IgD, IgG, IgM, HLA-DQ, HLA-DR, and IL6R) at 4°C for 30 min. Cells were washed once with PBS and 1% fetal bovine serum and were fixed with 1% paraformaldehyde. Data on cell-surface expression in each cell line were acquired using a fluorescence-activated cell sorter (BD Biosciences FACSCalibur system). To quantify expression for each LCL, we used flow cytometry, requiring at least 500 cells per LCL for it to be included in our analysis. Fluorescence intensity was measured for the anti-cell surface protein antibody and a control isotype antibody for each LCL. A marker (and, separately, a control) histogram was created by placing individual cell measurements into 1,024 equally spaced intensity bins. Counts in the control histogram were subtracted from the marker histogram to obtained a “normalized” histogram of cell-counts in each of the 1,024 intensity bins. The average intensity was then calculated from this normalized histogram and the log of this value was carried forward into QC as the average normalized marker expression for that LCL.

QC then proceeded by regressing this marker expression on the total cell count obtained for that marker within a given experimental batch of LCLs. (samples were batched by HapMap panel) We reasoned that if the experiment was successful, there should be no dependence of cell-surface marker expression on the quantity of viable cells obtained in the experiment; if there was such a dependence, the marker expression was likely reading out handling differences between LCLs, not true, intrinsic differences in expression. Indeed, by this metric, we found that during the first batch of experiments that was attempted (for the CEU panel), only 4 markers were successfully measured, while subsequent batches (YRI+CHB/JPT samples) succeeded for 14 and 9 markers respectively. In most markers that passed this filter, it was further noted that a few cell lines showed very low expression, far from the overall distribution of the values for each batch. While it is conceivable that these represent true differences, we interpreted these values as individual LCL measurement failures, and further truncated the lowest 5% of values within each marker in each batch. Thus, the final dataset contains measurements of: 85 cell lines for CD19 and CD20, 169 for CD21, 166 for CD227, 248 for CD40, 164 for CD58, 166 for CD80 and CD86, 248 for CD95, 80 for HLADQ, 85 for HLADR and IgM, and 165 for IgD, IgG, and IL6R. These values were centered within each panel and carried into further analysis.

### Luminex Assay

30 HapMap cell lines were screened with a multiplex antibody bead kit from Biosource (Cytokine 25-Plex for Luminex (Catalog #LHC0009)). Of the 25 cytokines originally selected for this assay, 8 were reliably detectable (lower concentration: IL8, IL10, IL12p40, TNFa, IP10; moderate concentration: MIP1a, MIP1b, RANTES). Of these, it was found that measurements for MIP1a and MIP1b were strongly correlated; thus we decided to include only MIP1b in further experiments. These 7 cytokines were assayed in the remainder of the cell lines according to the following protocol:

One cc for each LCL was placed into a single well of 96-deep well plate. The samples were centrifuged at 500 rpm for 5 minutes at room temperature. The supernatant was placed into a new 96-well plate, and placed dry ice to be stored at −80 degrees All assays were performed on a single thaw.

The cytokines were measured following the manufacturer's protocol. In order to ensure that the measured cytokine concentration fell in the linear part of the standard curve, the lower concentration cytokines were multiplexed together (final dilution 1∶2); and MIP1b and RANTES were multiplexed together (final dilution 1∶6).

The concentration of each cytokine was calculated based on the standard curve generated by the same plate, after subtracting out the “blank” background. A 3-parameter model was used to convert median fluorescent intensity (MFI) to protein concentration (ng/ml). A subsequent correction was applied to account for the dilution factor at the time of the assay. All final concentrations are expressed as pg/ml and log-transformed. 262 cell lines were successfully measured for IL10, IL12, IL8, IP10, and TNFa, and 266 measurements were obtained for MIP1b and RANTES. (79 and 87 biological replicate measurements were also obtained for the above two sets of cytokines respectively.)

### RNA Preparation and Affymetrix Expression Profiling

All LCLs were cultured in the fashion described above. Prior to the plating of cells for the Drug Response Assay, 5×10^5^ cells were set aside for RNA extraction. Cells were immediately lysed with Trizol Reagent (Invitrogen). RNA was collected according to the manufacturer instructions. 1.25 ug total RNA (OD>1.8) was diluted to a total volume of 10 uL. RNA was processed and hybridized onto Affymetrix Human U133A whole genome RNA expression genechip arrays according to the manufacturer's protocol. Gene expression summary values for the whole dataset were computed by RMA[40],[41] and log-transformed. Measurements were successfully obtained for 257 HapMap cell lines in the main experiment, for 64 biological replicates, for 24 cell lines originally thawed at the WTSI, as well as multiple replicates of 5 cell lines derived from chimpanzees. (Expression data is available on GEO Accession # GSE11582).

For analysis, the dataset was further processed as follows: 1) The ∼22 K total probe sets on the Affymetrics U133A were restricted to the 9084 judged expressed (p-value<0.06) by the Affymetrix software in at least 2/3 of 50 randomly selected scans. 2) These 9084 expressed probes were matched by Genbank transcript accession number (NM_#) to the 13,300 targets judged expressed by the same criterion in the WTSI Illumina HapMap experiments (using the probability of detection p-value output by the Illumina software.) This yielded a reduced set of 3600 Affymetrix probes (3592 Illumina targets) whose transcripts were reliably detectable in both experiments. 3) To obtain a comparable dataset from the WTSI Illumina data, we took the median over their 4 technical replicates for each target and quantile normalized across all samples. 4) We averaged within each gene symbol, in each dataset, for each sample, to get the set of 3538 genes expressed in both experiments and measured on both platforms. 5) To prevent family structure from introducing bias, the dataset was restricted to unrelated individuals only for the analyses in Figures 3–6: 198 each in the main Broad and WTSI experiments, 49 biological replicates at the Broad, and 16 samples for whom RNA was extracted at the WTSI and measured in both locations. Both centered (for each gene within each panel) and uncentered data is available in http://www.broad.mit.edu/mpg/pubs/hapmap_cell_lines/ and were each used as appropriate.

### Relative EBV and mtDNA Copy Number

All previously collected DNA was diluted to PCR concentration of 2.5 ng/uL and arrayed in 384 well storage plates (AbGene Cat No. AB-0564). Custom TaqMan assays were designed using Primer 3 (http://frodo.wi.mit.edu/) and ordered from Applied Biosystems. The EBV copy number assay interrogated a 66_bp fragment at the DNA polymerase locus (EBV forward primer 5′GACGA TCTTGGCAATCTCT3′, EBV reverse primer 5′TGGTCATGGATCTGCTAAACC3′, EBV probe 5′6FAM-CCACCTCCACGTGGATCACGA-MGBNFQ3′). The mtDNA copy number assay examined a 72 bp fragment at the ND2 locus (mtDNA forward primer TGTTGGTTATACCCTTCCCGTACTA, mtDNA reverse primer CCTGCAAAGATGGTAGAGTAGATGA, mtDNA probe sequence 5′6FAM-CCCTGGCCCAACCC-MGBNFQ3′).

As an internal reference, a 90 bp assay from the NRF1 locus on chromosome 7 was multiplexed with EBV or mtDNA (NRF1 forward primer 5′CTCGGTGTAAGTAGCCACAT 3′, NRF1 reverse primer 5′GAGTGACCCAAACCGAACAT 3′, NRF1 probe 5′VIC-CACTGCATGTGCTTCTATGGTAGCCA-MGBNFQ 3′). Equal efficiency of amplification was observed for each assay in the multiplex reaction. Final Concentrations for EBV primers, mtDNA primers, EBV probe, mtDNA probe, NRF1 primers and NRF1 probe were .25 uM, .25 uM, 10 uM, 10 uM, 1 uM and 10 uM respectively. 5 ng of DNA template was used for each TaqMan reaction performed according to the manufacturer's protocol. Relative EBV and mtDNA copy number was determined by the difference of CT method[42]. Log-transformed. EBV measurements were obtained when cell lines were first received from Coriell (257), during the main batch of experiments (257), and for the biological replicate set (86). Mitochondrial DNA measurements were obtained only for 252 cell lines in the main experiments.

### Fraction of RNA Variance Explained by Cellular Phenotype or eQTL (Figure 4)

We are interested in the fraction of gene-trait (or gene-eQTL) relationships that are real (i.e. would reach statistical significance given enough samples) and above a given r^2^ thresh-hold in the current sample. So, we want *P*(*real*, *r* ^2^ > = *c*) in joint distribution notation, i.e. a relationship can be real (non-null) or spurious (null) and can exceed a certain threshold or not. By regressing a trait on multiple genes, we observe: *P*(*r* ^2^ > = *c*). It is the fraction of relationships exceeding any given threshold, the green curve. By permutation, we also have: *P*(*r* ^2^ > = *c*|*not*_*real*), the blue (average of black) curve. So, we write, by conditioning on whether a relationship is real or not:
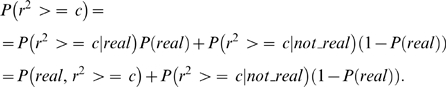
Or, rewriting, we have:
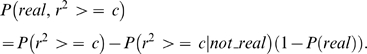
Everything on the right hand side is known, except P(real), the true proportion of gene-trait relationships in the data. This can theoretically be estimated ala Storey et al. 2003 [43] but the estimate can be unreliable in the setting of dependencies, as is the case in our data since genes are largely in clusters. So, we take the worst case scenario, setting P(real) = 0. Thus, we have:
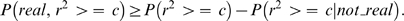
So, *P*(*r* ^2^ > = *c*)−*P*(*r* ^2^ > = *c*|*not*_*real*) is then a lower bound for *P*(*real*, *r* ^2^> = *c*), the black curve. It is important to note that the interpretation of this lower bound is limited to the sample size used in the analysis. Given more samples, the estimate will change to even more genes being affected by traits or eQTLs, albeit at lower r^2^s.

### Decomposing Gene Expression into Inter- and Intra-Components (Figure 5)

To estimate the amount of inter- and intra- individual variation present for each gene in the ∼50 unrelated individuals thawed and measured twice at the Broad Institute, we fit a random effects model of the form *y_ij_* = *μ*+*α_i_*+*ε_ij_*, where i indexes the individuals, and j is 1 or 2 for the biological replicate being considered. The estimated variance component 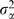 is then the inter-individual variation in gene expression for the gene, while the residual variance 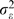 is the intra-individual variation. To evaluate the effect of a cis-eQTL or cellular phenotype on an RNA, a fixed effect x corresponding to trait was then added to the model to get: *y_ij_* = *μ*+*βx_ij_*+*α_i_*+*ε_ij_*. The resultant change in variance components 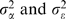 can then be interpreted as the “effect” of that trait or snp on RNA expression. The directionality of the effect is clearly only known for SNPs, but the nature of relationship (inter-, intra-, or both) can be examined for any trait. It's worth pausing to reflect on what these “effects” mean: If including a QTL SNP genotype in the model reduces inter-individual variance (as the overwhelming majority of SNPs do, Figure 5A), it implies that fixed differences in genotypes (QTLs) between individuals correlate to fixed differences in expression between individuals in the corresponding gene. (as one would expect) If, on the other hand, the intra-individual variance component is reduced when accounting for a given trait, the implication is that day-to-day variations in the trait correspond to day to day variations in the RNA. As would be expected, some genes also show a combination of the two effects. Finally, these estimates are quite noisy, suffering from random fluctuations in RNA levels, measurement error, and the relatively small sample size available for the analysis; estimation is likely even less reliable for weaker effects. Nevertheless, the analysis is instructive for the stronger signals and overall patterns and would improve given more samples and technical replicates.

### GWAS for Drug Response

1,045,141 autosomal SNPs with MAF>10% in each of the 3 (CEU, YRI, CHB/JPT) HapMap panels were selected from the Phase 2 HapMap build 21 for association testing to drug response phenotypes. The between/within family model of association was tested for each SNP against each drug, in each panel independently, using PLINK[32] v1.02 with options “–qfam-total –geno 1 –aperm 100 100 000 000 0.00000005 0.0001 5 0.001”. For each drug, p-values for each SNP were then combined across panels using Fisher's method. 25,735 X-chromosome SNPs were tested analogously, but using an additive model on unrelated individuals only with PLINK command line “–assoc –geno 1”; none exceeded 5e-8. QQ plots for the autosomal SNPs for each drug are available at: http://www.broad.mit.edu/mpg/pubs/hapmap_cell_lines/snps_vs_drug_response_pvalues/.

**R** – Aside from GWAS scans performed using PLINK, all other analyses were performed using R version 2.5.0[44].

## Supporting Information

Figure S1 Heritability power estimate. A power calculation was performed for the growth-rate and ATP level heritability estimates using the R package pwr (v1.1). Power to detect narrow-sense heritability (h^2^) is plotted as a function of heritability. The significance threshold (alpha) is set to 0.05. Sample sizes of the power calculation are set to the number of fully phenotyped trios used in the heritability estimates: N = 51 for ATP level and N = 37 for growth rate. The following assumptions were made in the power calculation: σ^2^_mother_ = σ^2^_father_ = σ^2^_offspring_ = 1; covariance(offspring, mother) = covariance(offspring, father); covariance(mother, father) = 0. Plot shows that the heritability estimates are not well-powered to detect heritability<0.5.
(0.01 MB PDF)

Figure S2 Drug response GWAS power estimate. A power calculation was performed for the drug response GWAS using the R package pwr (v1.1). Power to discover a QTL is plotted as a function of the fraction of variance in drug response the putative QTL explains. The significance threshold (alpha) is set to the genome-wide significance level of 5e-8. As the GWAS was performed in trios, two estimates are plotted: (1) a lower bound on power corresponding to the scan including only successfully measured unrelated individuals (i.e., no useful information from trio kids has been derived); and (2) an upper bound on power corresponding to trio kids providing as much information as another unrelated individual. As trio kids actually provide an intermediate amount of extra information, true power of the study lies between the two bounds. Plot shows that the GWAS is only well-powered to detect strong (>15% variance explained) drug response QTLs.
(0.01 MB PDF)

Figure S3 Direction of SNP-Drug response association. For each tuple (WTSI – red, Broad – green) in Figure 6, the product of the correlation (r) between SNP and RNA and the correlation (rho) between RNA and Drug is plotted against the correlation (r) between SNP and Drug. Black lines separate the plot into the 4 quadrants. Gray dotted lines show the expected distribution of associations between SNP and Drug under the “null” model simulated in Figure 6B. Plot shows that the direction of association SNP-Drug response tends toward the direction predicted from the directions of the SNP-RNA and RNA-Drug correlations (i.e., if the major allele drives the RNA up and more RNA makes the cell-line more sensitive to drug, then the major allele should make the cell-line more sensitive to drug). This tendency would not be expected by chance alone.
(0.01 MB PDF)

Figure S4 Winner's curse in eQTL discovery. Simulations were performed to demonstrate that effect sizes of weaker eQTLs are overestimated, on average. Specifically, for effect sizes (r^2^) between 0.01 and 0.50, 100,000 datasets of 198 values each (corresponding to the sample size of the analysis in Fig. 6) were simulated from a bivariate normal distribution with mean = (0,0), variances = (1,1) and covariances = sqrt(effect size). Datasets with observed correlation (r^2^)>0.08 were then considered: For each simulated effect sizes, the average difference (bias) between the observed and simulated effect size is plotted, together with the standard deviation of the distribution of differences. Plot shows that weaker eQTLs are usually over-estimated, even for true effects that are above the detection threshold. On the other hand, estimates of effect sizes of stronger eQTLs are unbiased, on average.
(0.14 MB PDF)

Table S1 Correlation between relative drug responses on replicate plates.
(0.13 MB PDF)

Table S2 Correlation between relative drug responses in independent experiments.
(0.12 MB PDF)

Table S3 Correlation between relative drug responses and growth rates.
(0.17 MB PDF)

Table S4 Correlation between growth-rate corrected EC50s for each of the drugs and growth rates.
(0.17 MB PDF)

Table S5 Correlation between growth-rate and ATP-corrected EC50s for each of the drugs.
(0.15 MB PDF)
