# Supplementary figures and images for: Genetic Analysis of Human Traits In Vitro: Drug Response and Gene Expression in Lymphoblastoid Cell Lines
Source: PLoS Genet. 2008 Nov 28;4(11):e1000287. doi: 10.1371/journal.pgen.1000287 (PMC2583954; doi:10.1371/journal.pgen.1000287)

## Heritability power estimate

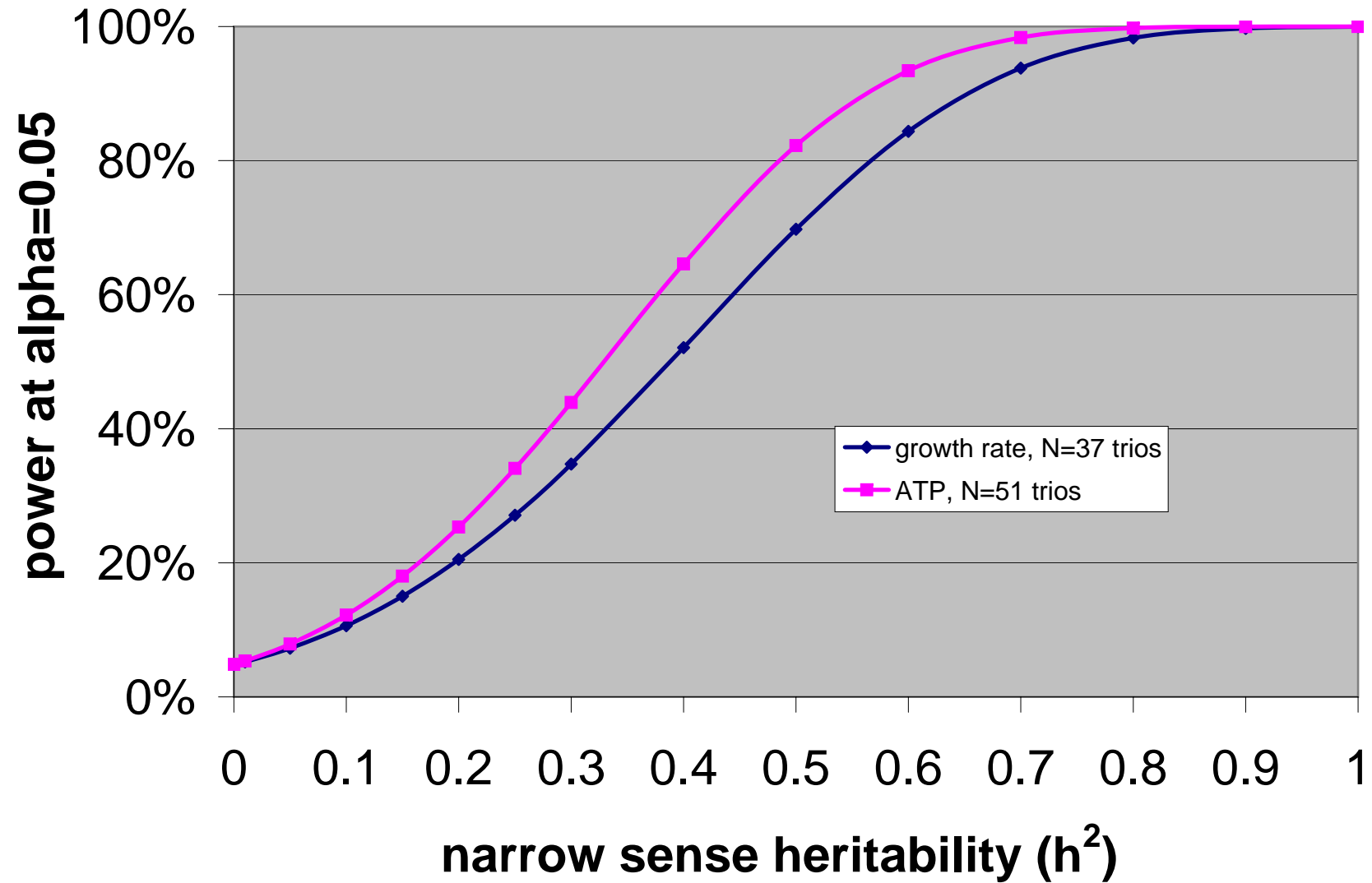

Supplement: Figure S1 — Heritability power estimate. A power calculation was performed for the growth-rate and ATP level heritability estimates using the R package pwr (v1.1). Power to detect narrow-sense heritability (h2) is plotted as a function of heritability. The significance threshold (alpha) is set to 0.05. Sample sizes of the power calculation are set to the number of fully phenotyped trios used in the heritability estimates: N = 51 for ATP level and N = 37 for growth rate. The following assumptions were made in the power calculation: σ2 mother = σ2 father = σ2 offspring = 1; covariance(offspring, mother) = covariance(offspring, father); covariance(mother, father) = 0. Plot shows that the heritability estimates are not well-powered to detect heritability<0.5. (0.01 MB PDF) [file pgen.1000287.s001.pdf]

## Drug response GWAS power estimate

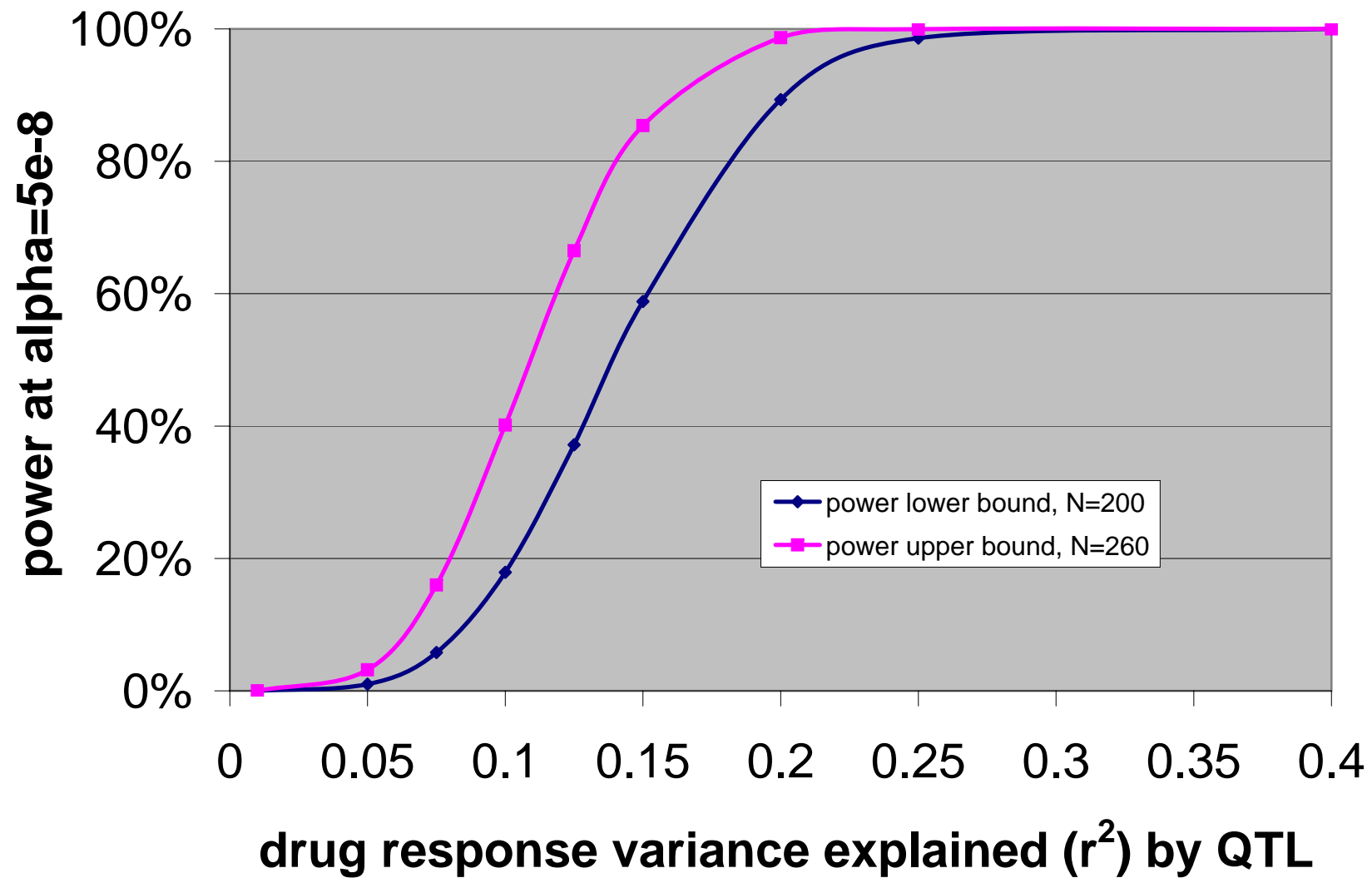

Supplement: Figure S2 — Drug response GWAS power estimate. A power calculation was performed for the drug response GWAS using the R package pwr (v1.1). Power to discover a QTL is plotted as a function of the fraction of variance in drug response the putative QTL explains. The significance threshold (alpha) is set to the genome-wide significance level of 5e-8. As the GWAS was performed in trios, two estimates are plotted: (1) a lower bound on power corresponding to the scan including only successfully measured unrelated individuals (i.e., no useful information from trio kids has been derived); and (2) an upper bound on power corresponding to trio kids providing as much information as another unrelated individual. As trio kids actually provide an intermediate amount of extra information, true power of the study lies between the two bounds. Plot shows that the GWAS is only well-powered to detect strong (>15% variance explained) drug response QTLs. (0.01 MB PDF) [file pgen.1000287.s002.pdf]

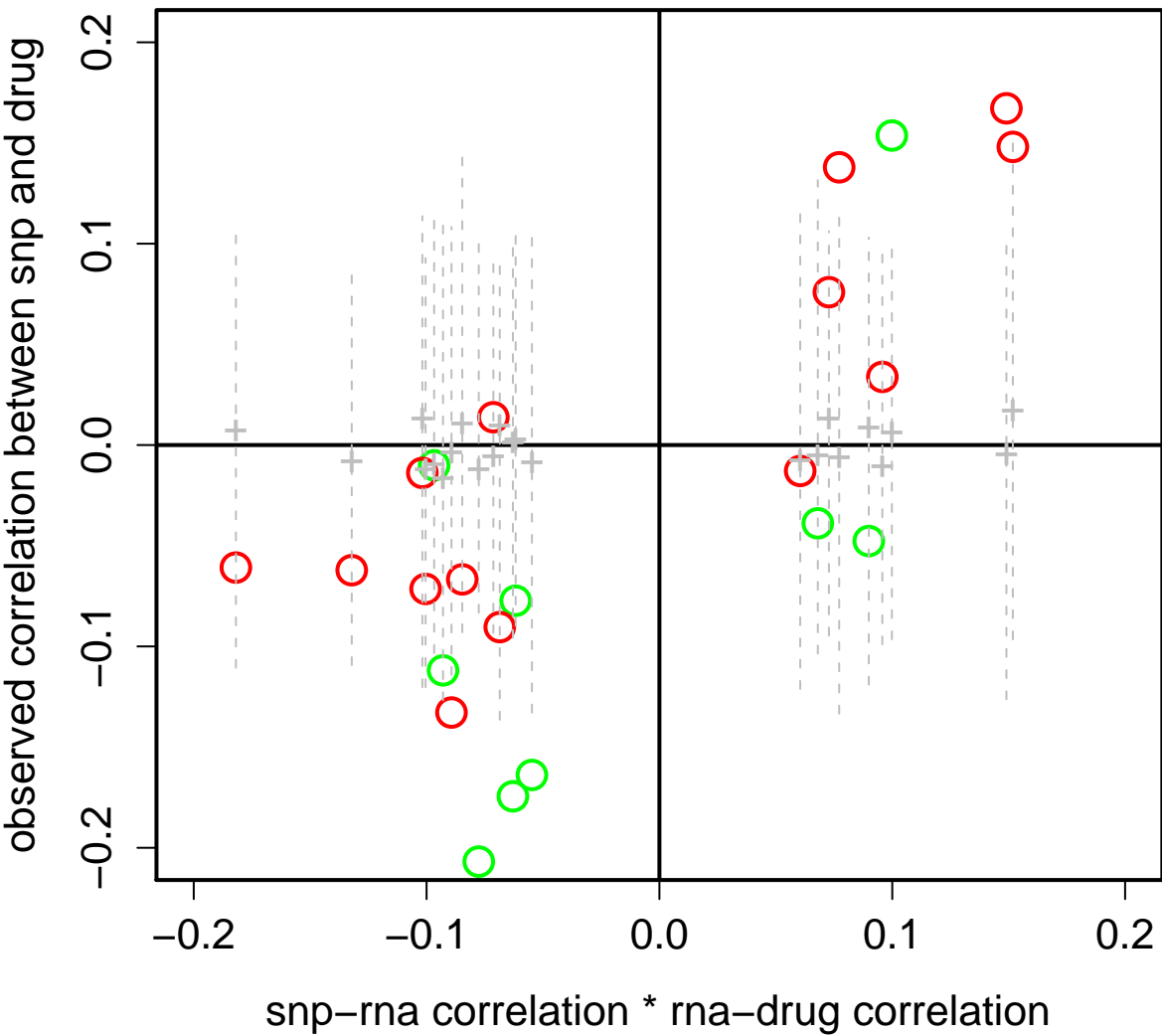

Supplement: Figure S3 — Direction of SNP-Drug response association. For each tuple (WTSI – red, Broad – green) in Figure 6, the product of the correlation (r) between SNP and RNA and the correlation (rho) between RNA and Drug is plotted against the correlation (r) between SNP and Drug. Black lines separate the plot into the 4 quadrants. Gray dotted lines show the expected distribution of associations between SNP and Drug under the “null” model simulated in Figure 6B. Plot shows that the direction of association SNP-Drug response tends toward the direction predicted from the directions of the SNP-RNA and RNA-Drug correlations (i.e., if the major allele drives the RNA up and more RNA makes the cell-line more sensitive to drug, then the major allele should make the cell-line more sensitive to drug). This tendency would not be expected by chance alone. (0.01 MB PDF) [file pgen.1000287.s003.pdf]

# Winner's curse: 198 samples, detection at $r^2=0.08$

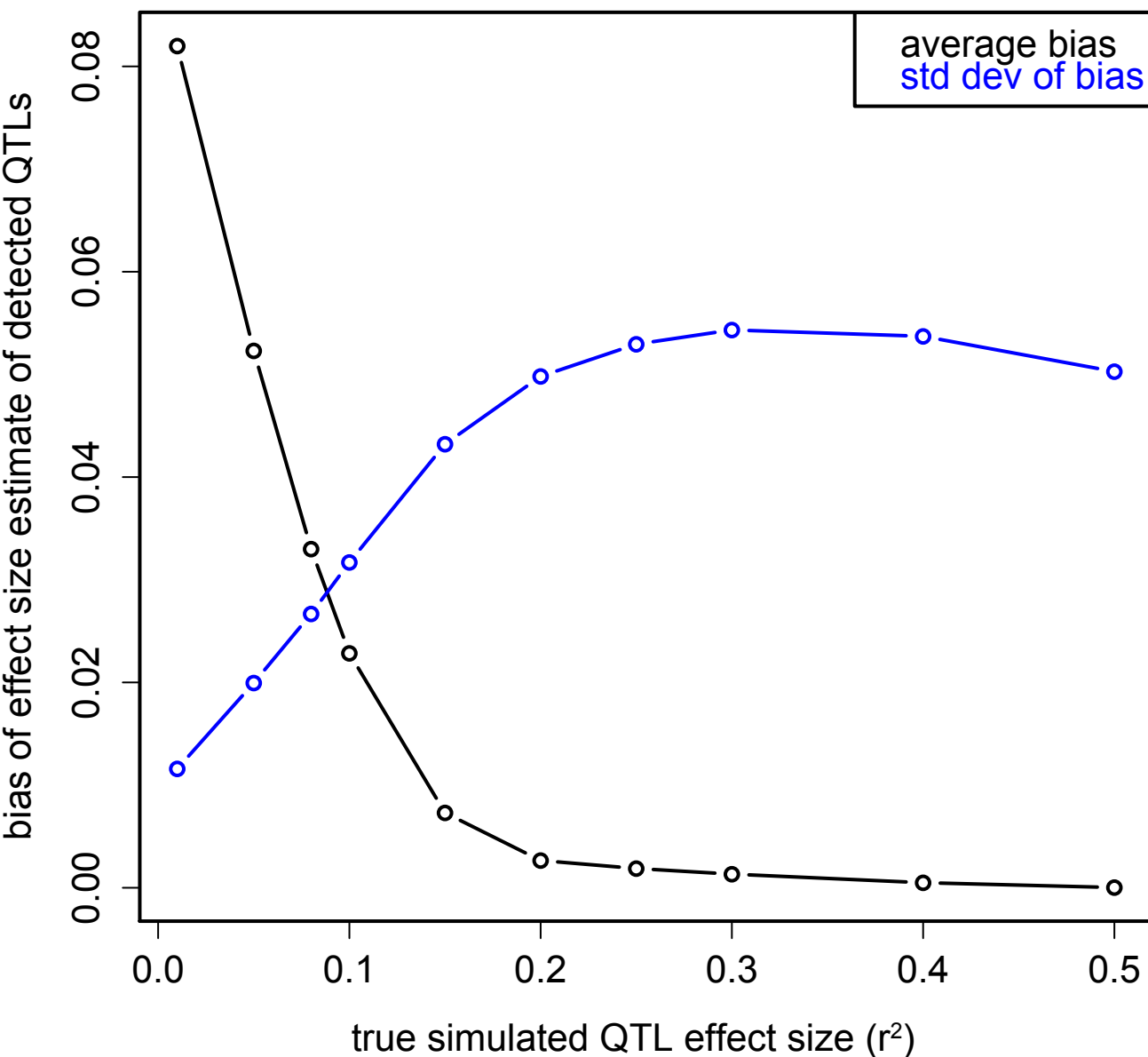

Supplement: Figure S4 — Winner's curse in eQTL discovery. Simulations were performed to demonstrate that effect sizes of weaker eQTLs are overestimated, on average. Specifically, for effect sizes (r2) between 0.01 and 0.50, 100,000 datasets of 198 values each (corresponding to the sample size of the analysis in Fig. 6) were simulated from a bivariate normal distribution with mean = (0,0), variances = (1,1) and covariances = sqrt(effect size). Datasets with observed correlation (r2)>0.08 were then considered: For each simulated effect sizes, the average difference (bias) between the observed and simulated effect size is plotted, together with the standard deviation of the distribution of differences. Plot shows that weaker eQTLs are usually over-estimated, even for true effects that are above the detection threshold. On the other hand, estimates of effect sizes of stronger eQTLs are unbiased, on average. (0.14 MB PDF) [file pgen.1000287.s004.pdf]
