# Supplementary material for: Genetic Analysis of Human Traits In Vitro: Drug Response and Gene Expression in Lymphoblastoid Cell Lines
Source: PLoS Genet. 2008 Nov 28;4(11):e1000287. doi: 10.1371/journal.pgen.1000287 (PMC2583954; doi:10.1371/journal.pgen.1000287)
Supplement: Table S1 — Correlation between relative drug responses on replicate plates. (0.13 MB PDF) [file pgen.1000287.s005.pdf]

Compound	rho	rho^2	pval
MTX	0.98831	0.976749	<2.20e-16
5FU	0.97217	0.945105	<2.20e-16
6MP	0.94395	0.891043	<2.20e-16
Simva	0.91629	0.839582	<2.20e-16
Saha	0.85846	0.736949	<2.20e-16
velcade	0.88812	0.788753	<2.20e-16
rapa	0.91968	0.845818	<2.20e-16
