# Supplementary material for: Genetic Analysis of Human Traits In Vitro: Drug Response and Gene Expression in Lymphoblastoid Cell Lines
Source: PLoS Genet. 2008 Nov 28;4(11):e1000287. doi: 10.1371/journal.pgen.1000287 (PMC2583954; doi:10.1371/journal.pgen.1000287)
Supplement: Table S2 — Correlation between relative drug responses in independent experiments. (0.12 MB PDF) [file pgen.1000287.s006.pdf]

Compound	rho	rho^2	pval
MTX	0.82	0.67	1.2E-15
5FU	0.63	0.39	6.6E-08
6MP	0.66	0.43	9.7E-09
Simva	0.39	0.15	2.0E-03
Saha	0.71	0.50	2.0E-10
