# Supplementary material for: Genetic Analysis of Human Traits In Vitro: Drug Response and Gene Expression in Lymphoblastoid Cell Lines
Source: PLoS Genet. 2008 Nov 28;4(11):e1000287. doi: 10.1371/journal.pgen.1000287 (PMC2583954; doi:10.1371/journal.pgen.1000287)
Supplement: Table S3 — Correlation between relative drug responses and growth rates. (0.17 MB PDF) [file pgen.1000287.s007.pdf]

Relative Drug Response	MTX	6MP	5FU	Simva	Saha
MTX	*rank correlation*	2.20E-16	2.20E-16	1.14E-08	0.00977
6MP	0.78	*below*	2.20E-16	7.44E-05	0.00127
5FU	0.78	0.61	*diagonal,*	4.68E-14	1.26E-06
Simva	0.36	0.25	0.46	*p-value*	1.97E-04
Saha	0.16	0.2	0.3	0.23	*above*

pvalues <.001 marked in red

Correlation to Growth Rate of relative drug response

Drug	MTX	6MP	5FU	Simva	Saha
Rank Correlation	-0.34	-0.31	-0.3	-0.15	0.04
P-value	1.86E-07	3.21E-06	7.61E-06	0.031	0.603

pvalues <.001 marked in red
