# Supplementary material for: Genetic Analysis of Human Traits In Vitro: Drug Response and Gene Expression in Lymphoblastoid Cell Lines
Source: PLoS Genet. 2008 Nov 28;4(11):e1000287. doi: 10.1371/journal.pgen.1000287 (PMC2583954; doi:10.1371/journal.pgen.1000287)
Supplement: Table S4 — Correlation between growth-rate corrected EC50s for each of the drugs and growth rates. (0.17 MB PDF) [file pgen.1000287.s008.pdf]

**Growth-corrected EC50 Drug Response**	**MTX**	**6MP**	**5FU**	**Simva**	**Saha**
**MTX**	*rank correlation*	0.02	1.74E-09	2.27E-11	0.174
**6MP**	0.14	*below*	0.0165	0.973	0.0524
**5FU**	0.37	0.15	*diagonal,*	0.00863	1.54E-04
**Simva**	0.41	0.0021	0.17	*p-value*	0.118
**Saha**	0.09	0.12	0.24	0.1	*above*

pvalues <.001 marked in red

Correlation to Growth Rate of EC50/Growth-corrected drug responses

Drug	**MTX**	**6MP**	**5FU**	**Simva**	**Saha**
Rank Correlation	-0.1	0.06	-0.24	-0.14	0.06
P-value	0.14	0.4	0.00034	0.0356	0.345

pvalues <.001 marked in red
