# Supplementary material for: Genetic Analysis of Human Traits In Vitro: Drug Response and Gene Expression in Lymphoblastoid Cell Lines
Source: PLoS Genet. 2008 Nov 28;4(11):e1000287. doi: 10.1371/journal.pgen.1000287 (PMC2583954; doi:10.1371/journal.pgen.1000287)
Supplement: Table S5 — Correlation between growth-rate and ATP-corrected EC50s for each of the drugs. (0.15 MB PDF) [file pgen.1000287.s009.pdf]

Growth and ATP-Corrected Drug Response	MTX	6MP	5FU	Simva	Saha
**MTX**	*rank correlation*	0.041	0.008	2.06E-08	0.703
**6MP**	0.15	*below*	0.003	0.973	0.052
**5FU**	0.19	0.22	*diagonal,*	0.291	0.006
**Simva**	0.40	0.00	0.08	*p-value*	0.118
**Saha**	-0.03	0.12	0.20	0.10	*above*

pvalues <.001 marked in red
